# Tissue-Specific Enhancement of Insulin Function and Restoration of Glucose-Stimulated Insulin Secretion by *Croton guatemalensis* Lotsy and *Eryngium cymosum* F. Delaroche

**DOI:** 10.3390/ph18101433

**Published:** 2025-09-24

**Authors:** Fernanda Artemisa Espinoza-Hernández, Angelina Daniela Moreno-Vargas, Andrea Díaz-Villaseñor, Gerardo Mata-Torres, Jazmín Samario-Román, Adolfo Andrade-Cetto

**Affiliations:** 1 Departamento de Biología Celular, Facultad de Ciencias, Universidad Nacional Autónoma de México (UNAM), Ciudad Universitaria, Coyoacán, Mexico City 04510, Mexico; f.artemisa.ehdz@ciencias.unam.mx (F.A.E.-H.);; 2 Posgrado en Ciencias Biológicas, Unidad de Posgrado, Universidad Nacional Autónoma de México (UNAM), Edificio D, 1° Piso, Circuito de Posgrados, Ciudad Universitaria, Coyoacán, Mexico City 04510, Mexico; 3 Departamento de Medicina Genómica y Toxicología Ambiental, Instituto de Investigaciones Biomédicas, Universidad Nacional Autónoma de México (UNAM), Ciudad Universitaria, Coyoacán, Mexico City 04510, Mexico

**Keywords:** medicinal plants, *Croton guatemalensis*, *Eryngium cymosum*, insulin resistance, Akt, glucose-stimulated insulin secretion

## Abstract

**Background/Objectives**: Ethnopharmacological studies indicates that plant-based infusions are usually consumed by some people in advanced stages of diabetes, that is, when poor pancreatic dysfunction coexists with insulin resistance (IR). Current treatments aim to prevent β-cell deterioration by promoting improved insulin function and/or enhancing pancreatic function to avoid the development of hyperglycemia. Therefore, *Croton guatemalensis* (Cg) and *Eryngium cymosum* (Ec), two medicinal plants with potential insulin-sensitizing effects described in previous studies, were assessed on parameters related to IR and on the architecture of pancreatic islets in rats exposed to a syrup containing 8.8% glucose and 5.2% fructose in drinking water. **Methods**: After an 8-week exposure to syrup, plant extracts were orally administered for four weeks at traditional doses (Cg: 30 mg/kg body weight; Ec: 470 mg/kg body weight). Body weight, food intake, and drinking water consumption were monitored. At the end of the study, IR surrogate indices were calculated, metabolic assays were performed, and white adipose tissues, liver, gastrocnemius muscle, and pancreas were extracted in fasting and postprandial state for lipid quantification (liver), measurement of Akt phosphorylation status by western blot (liver and muscle), and determination of insulin content by immunohistochemistry (pancreatic islets). **Results**: Both species decreased hepatic lipid content without promoting significant changes in visceral adiposity. Although they did not improve surrogate markers of fasting IR, both ameliorated insulin function, glucose tolerance, and restored the glucose-stimulated insulin secretory response in metabolic tests. Cg restored the insulin signaling response in liver and muscle, whereas Ec only did so in muscle. Moreover, both appeared to enhance insulin pancreatic content or restore pancreatic islet population. **Conclusions**: Cg and Ec can reverse the IR phenotype in a tissue-specific manner and improve pancreatic function.

## 1. Introduction

Insulin resistance (IR) is a hallmark of several metabolic diseases, including metabolic syndrome, obesity, type 2 diabetes (T2D), metabolic-dysfunction-associated fatty liver disease, cardiovascular disease, and polycystic ovary syndrome. It can be defined as a pathophysiological condition in which insulin-sensitive target tissues do not perform their functions adequately in the presence of physiological levels of the hormone insulin. Therefore, abnormalities in carbohydrate and lipid metabolism may occur, such as exacerbated hepatic glucose production, decreased peripheral glucose uptake, and increased lipolysis [1,2].

It is widely accepted that increased adiposity is the direct cause of IR. Low-grade inflammation caused by adipocyte hypertrophy promotes impairment of the insulin signaling pathway by increasing phosphorylation on serine residues rather than tyrosine residues and by promoting the degradation and dephosphorylation of various components of the pathway. This results in a downregulation of protein kinase B (Akt), the master regulator of metabolism [3,4].

To compensate IR, pancreatic β cells secrete increased amounts of insulin into the bloodstream. Consequently, one of the characteristic signs of this pathology is the presence of hyperinsulinemia. In this sense, it is said that there is a balance between the demand for insulin by its target organs and the insulin secreted by pancreatic β cells to maintain glucose homeostasis [5]. In a context of IR, this balance is disrupted, resulting in increased insulin secretion to counteract the poor tissue function required to maintain euglycemia. If IR is not reversed through the implementation of lifestyle changes, basically nutrition improvement and physical exercise, pancreatic β-cell failure may occur due to the prolonged maintenance of hyperinsulinemia to compensate for poor insulin function. At this point, hyperglycemia begins to become evident and progresses to T2D [2,6].

Currently, there is no drug that directly improves insulin function by affecting the insulin signaling pathway. Biguanides such as metformin (Met) and thiazolidinediones such as pioglitazone improve systemic insulin sensitivity indirectly by reducing low-grade inflammation and adiposity or by indirectly modulating the pathway [7,8]. Moreover, both drugs only have a positive effect on insulin function without having a direct impact on β cell, while current therapies aim to both promote insulin sensitization and restore pancreatic function [9]. Therefore, the search continues for molecules that can exert a direct positive impact on insulin signaling and β-cell function, with the goal of preventing the progression of metabolic diseases such as diabetes and cardiovascular disease.

The use of medicinal plants to treat diseases is a common practice around the world. Particularly, there is growing evidence for their use in the treatment of diabetes, i.e., to counteract blood “sugar” levels. In this context, patients begin consuming plant species after a medical diagnosis that usually occurs when the disease is in its advanced stages and hyperglycemia levels are potentially uncontrolled. Previously, we have documented that the late implementation of treatment with medicinal plants at traditional doses is able to prevent the worsening of the hyperglycemic condition, avoiding the increase in glycated hemoglobin [10]. Therefore, the next research question is whether intervention with medicinal plants in early stages can reverse the prediabetic condition. For this purpose, two medicinal plants traditionally used in Central American and Mexican medicine were evaluated for their effects on metabolic parameters associated with IR in rats exposed to a glucose–fructose syrup solution in drinking water, a model whose detrimental impact on insulin function and glucose and lipid metabolism is well documented in the literature [11,12,13].

*Croton guatemalensis* Lotsy (Euphorbiaceae) (Cg) and *Eryngium cymosum* F. Delaroche (Apiaceae) (Ec) are species traditionally used for the treatment of diabetes by the Cakchiquel community of Guatemala and by patients from the municipalities of Tlanchinol and Huejutla de Reyes, Hidalgo, Mexico. Cg, commonly known as “copalchí”, is a small tree, up to 6 m high, distributed in the tropical and subtropical areas of the Americas. On the other hand, Ec, also known as “piñuela”, is a robust perennial herb distributed in Mexico and Guatemala. The infusion of both species is prepared by boiling a handful (20 g) of the plant (Cg: bark; Ec: aerial parts) in half a liter of water and is consumed throughout the day. In previous works, they have demonstrated the ability to acutely regulate blood glucose levels in fasting and postprandial states in streptozotocin (STZ)-induced hyperglycemic rats, where one of the mechanisms implied was the improvement of insulin sensitivity [14,15,16]. In addition, the phytochemical composition of both species has been previously reported: the main compounds of Cg are *ent*-clerodane diterpenoids and phenolic acids have been identified in Ec. Specifically, the major compound of the ethanol–water extract of Cg is junceic acid, while, for the aqueous extract of Ec, it is rosmarinic and chlorogenic acids [17,18]. Thus, we aimed to assess the impact of the chronic administration of both extracts at traditional doses on metabolic parameters related to IR, evaluating Akt phosphorylation activation status between fasting and postprandial states in two of the major insulin target organs, liver and muscle. On the other hand, the impact of chronic treatment on pancreatic islet architecture was also evaluated to gain an overview of the effect of the extracts of these two medicinal plants on insulin secretion and pancreatic function.

## 2. Results

### 2.1. Changes in Weight Gain, Calorie Intake, and Body Composition

**Figure 1:**
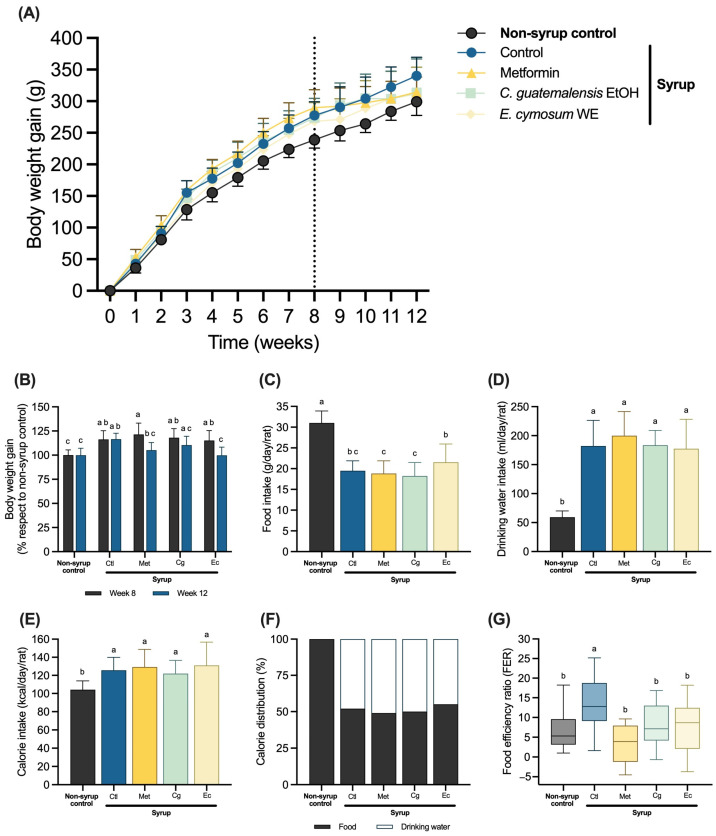
Body weight gain and calorie consumption: (**A**) Body weight gain during the 12-week experiment. The dotted line indicates the beginning of the oral treatments. (**B**) Body weight gain from week 8 to week 12 expressed as a percentage of the non-syrup control. Food consumption (**C**), drinking water intake (**D**), calorie ingestion (**E**), and calorie distribution (**F**) during the treatment period (weeks 8 to 12), (**G**) FER values. Data are expressed as mean ± SD (*n* = 8). One-way ANOVA followed by Tukey post hoc tests were applied to compare the means among groups. Different letters over the bars indicate statistically significant differences (a > b > c), *p* < 0.05. Ctl, syrup control; Met, syrup group treated with metformin (850 mg/kg b.w.); Cg, syrup group treated with *C. guatemalensis* extract (30 mg/kg b.w.); Ec, syrup group treated with *E. cymosum* extract (470 mg/kg b.w.).

Rats fed syrup were more likely to increase their body weight (b.w.) from the first week of syrup intake, being more evident by the fourth week (Figure 1A). At week eight, syrup rats showed a significant increase in weight compared with non-syrup rats (Figure 1B). At the end of the experiment, only weight gain from syrup control, which was given a physiological solution, was statistically different compared with that from the non-syrup control. Groups receiving Met and Ec significantly reduced b.w. gain relative to their own b.w. at week eight, resembling the non-syrup control (Figure 1B). Regardless of the treatments administered via gavage, all syrup groups significantly decreased their food intake and increased their drinking water intake compared with the non-syrup control (Figure 1C,D), leading to a significant rise in total calorie consumption that was similar among all syrup groups (Figure 1E), also with a comparable proportion of calories derived from food and drink across the four groups (Figure 1F). Although none of the treatments modified total caloric intake, food efficiency ratio (FER; b.w. gain/food intake per day), which was significantly higher in the syrup control than in the non-syrup control, was significantly lowered by Met and extracts (Figure 1G).

**Figure 2:**
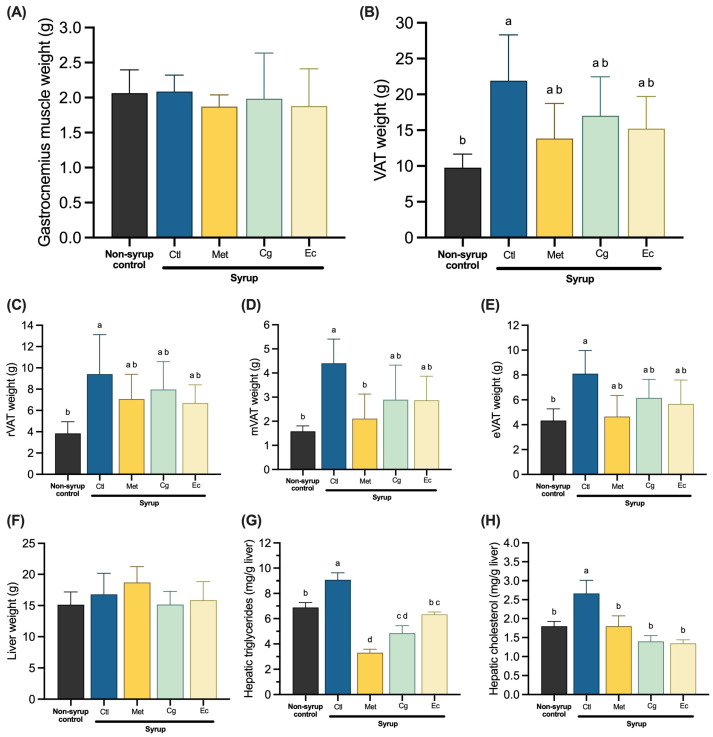
Tissue weight and hepatic lipid content in fasting: (**A**) Weight of gastrocnemius muscle. (**B**) Weight of VAT (retroperitoneal, mesenteric, and epididymal tissues). (**C**) Weight of rVAT. (**D**) Weight of mVAT. (**E**) Weight of eVAT. (**F**) Weight of liver. Data are expressed as mean ± SD (*n* = 4). (**G**) Levels of hepatic triglycerides. (**H**) Levels of hepatic total cholesterol. Data are expressed as mean ± SEM (*n* = 4). One-way ANOVA followed by Tukey post hoc tests were applied to compare the means among groups. Different letters over the bars indicate statistically significant differences (a > b > c > d), *p* < 0.05. Ctl, syrup control; Met, syrup group treated with metformin (850 mg/kg b.w.); Cg, syrup group treated with *C. guatemalensis* extract (30 mg/kg b.w.); Ec, syrup group treated with *E. cymosum* extract (470 mg/kg b.w.).

Chronic syrup consumption promoted significant changes in the amount of visceral white adipose tissue (VAT) without altering gastrocnemius muscle weight (Figure 2A,B). The syrup control group increased VAT weight 2.25 times compared with the non-syrup control group. This rise was partially prevented by Met and extract treatments, whose VAT weight was also statistically similar to that of the control without syrup (Figure 2B). When analyzing VAT depots separately, it was observed that Met and Ec did not significantly prevent the increase in retroperitoneal (rVAT) weight tissues (Figure 2C), while only Met was able to significantly decrease mesenteric (mVAT) weight tissue (Figure 2D). On the other hand, no treatment promoted significant changes in epididymal (eVAT) weight tissue, showing only a tendency to decrease it (Figure 2E). Regarding the weight of livers, there were no differences among the experimental groups (Figure 2F). However, hepatic triglyceride and cholesterol levels were significantly reduced by Met and both plant extracts compared with syrup control, indicating a restoration of hepatic lipid metabolism altered by syrup consumption (Figure 2G,H).

All these results suggest that four-week treatment with Met and both extracts decreases hepatic lipid content without exerting significant changes in VAT, while only Met and Ec reduce b.w. gain promoted by chronic syrup consumption.

### 2.2. Surrogate Markers of Fasting Insulin Sensitivity

**Figure 3:**
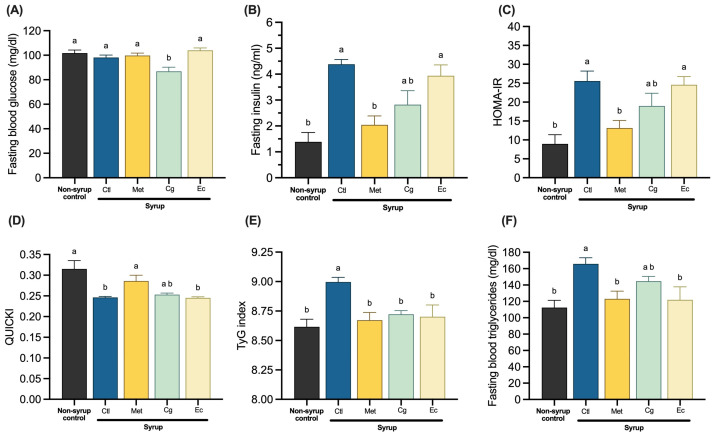
Fasting insulin sensitivity: (**A**) Fasting blood glucose. (**B**) Fasting insulin. (**C**) HOMA-IR. (**D**) QUICKI. (**E**) TyG index. (**F**) Fasting blood triglycerides. Data are expressed as mean ± SEM (*n* = 8). One-way ANOVA followed by Tukey post hoc tests were applied to compare the means among groups. Different letters over the bars indicate statistically significant differences (a > b), *p* < 0.05. Ctl, syrup control; Met, syrup group treated with metformin (850 mg/kg b.w.); Cg, syrup group treated with *C. guatemalensis* extract (30 mg/kg b.w.); Ec, syrup group treated with *E. cymosum* extract (470 mg/kg b.w.).

Chronic syrup consumption did not produce significant changes in fasting blood glucose (FBG) levels. Nevertheless, only treatment with Cg statistically decreased this parameter (Figure 3A). When analyzing fasting insulin (FI), it was observed that syrup intake promoted a significant hyperinsulinemia denoting IR, which was completely abolished by Met treatment, as expected (Figure 3B). Interestingly, Ec treatment did not decrease the hyperinsulinemic state, whereas Cg treatment showed a tendency to reduced it (*p* = 0.0953). Treatments with both plants replicated this behavior when analyzing the surrogate indices that relate glucose metabolism to insulin, the homeostatic model assessment of IR (HOMA-IR), and the quantitative insulin sensitivity check index (QUICKI), where a significant decrease and increase were observed, respectively, in the group treated with Met (Figure 3C,D). These results suggest that the Cg extract may be less effective than Met in improving insulin sensitivity during fasting, while the Ec extract showed no impact at all, as it did not even reduce FI levels. However, triglyceride/glucose (TyG) index, another surrogate IR marker that considers FBG and fasting blood triglyceride (FBT) levels [19], showed that both Ec and Cg extracts decreased the levels of this index, implying an effect on IR (Figure 3E). In spite of that, Ec significantly was able to reduce hypertriglyceridemia caused by syrup intake, whereas Cg only tented to show a small trend (Figure 3F).

### 2.3. Dynamic Insulin Sensitivity Tests

**Figure 4:**
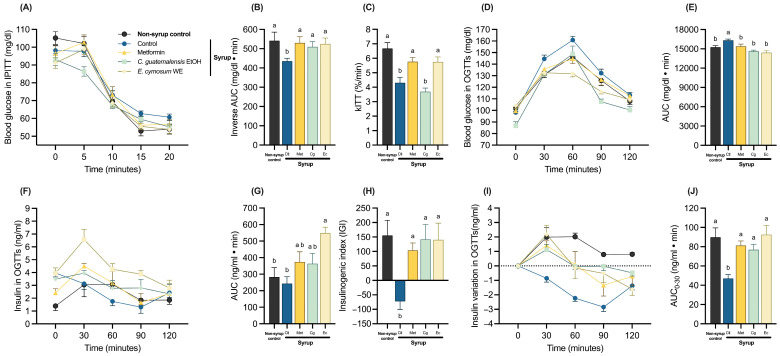
Metabolic challenges assessing insulin sensitivity and β-cell function: (**A**) Intraperitoneal insulin tolerance tests. (**B**) Inverse AUC of intraperitoneal insulin tolerance tests. (**C**) Rate of glucose disappearance constant (kITT) of intraperitoneal insulin tolerance tests. (**D**) Oral glucose tolerance tests. (**E**) AUC of oral glucose tolerance tests. (**F**) Insulin levels during oral glucose tolerance tests. (**G**) Insulin AUC during oral glucose tolerance tests. (**H**) Insulinogenic index obtained from glucose and insulin values at times 0 and 30. (**I**) Insulin variation during oral glucose tolerance tests. (**J**) Insulin AUC calculated at times 0 and 30 of the insulin variation. Data are expressed as mean ± SEM (*n* = 8). One-way ANOVA followed by Tukey post hoc tests were applied to compare the means among groups. Different letters over the bars indicate statistically significant differences (a > b), *p* < 0.05. Ctl, syrup control; Met, syrup group treated with metformin (850 mg/kg b.w.); Cg, syrup group treated with *C. guatemalensis* extract (30 mg/kg b.w.); Ec, syrup group treated with *E. cymosum* extract (470 mg/kg b.w.).

Given the ambiguous results obtained regarding the effect of the extracts on fasting insulin resistance/sensitivity, dynamic tests were performed to assess glucose regulation through a metabolic challenge, such as a glucose or insulin load, since blood glucose is tightly regulated not only by insulin sensitivity, but also by β-cell function [20]. The intraperitoneal insulin tolerance tests (IPITTs) showed that Met and both extracts exacerbated the hypoglycemic effect of insulin, which was reflected in a significant increase in total glucose uptake from the bloodstream as seen in the inverse area under the curve (AUC) compared with the group treated with syrup alone (Figure 4A,B). Furthermore, only Met and Ec treatments augmented the rate of glucose uptake that was decreased by syrup consumption (Figure 4C).

On the other hand, glucose intolerance promoted by syrup solution was improved by treatment with Met and both extracts, resulting in a decrease in the AUC of oral glucose tolerance tests (OGTT) (Figure 4D,E). Although Cg was likely to promote a more evident decrease at 90 and 120 min, Ec had a marked antihyperglycemic effect, avoiding the hyperglycemic peak at 60 min followed by a gradual decrease in blood glucose levels. When assessing insulin levels during the OGTT, a loss of glucose-stimulated insulin secretion (GSIS) in the syrup-treated rats was appreciated (Figure 4F), despite exhibiting the same insulin AUC as the non-syrup control (Figure 4G). Moreover, although groups treated with Met and both extracts exhibited higher insulin levels throughout the entire test, with Ec exhibiting significantly higher levels than the other groups, they were able to recover the GSIS response (Figure 4H,I). Accordingly, all treatments restored the insulin AUC from the first 30 min of the test (Figure 4J).

Altogether, these findings suggest that, despite not showing improvement over surrogate markers of fasting insulin sensitivity such as Met, both Cg and Ec treatments are capable of regulating glucose homeostasis through the improvement of insulin function and/or glucose intolerance, as well as GSIS restoration. Hence, the following experiments aimed to delve deeper into the tissue-specific effect of the extracts on the insulin signaling pathway in liver and muscle, as well as assessing the effect on pancreatic islets.

### 2.4. Tissue-Specific Insulin Function

Under normal physiological conditions, insulin secreted by β-cells in response to nutrients binds to its receptor on its target organs, activating the signaling pathway that leads to the stimulation of Akt and the subsequent anabolic response in the postprandial state. Therefore, the phosphorylation of this master metabolism regulator is a marker denoting proper insulin function [21]. To specifically assess the impact of the extracts on IR, the abundance of the Akt protein in liver and muscle was evaluated, as well as the phosphorylation status of two of its most representative isoforms: Akt1 and Akt2. Akt1 is ubiquitously expressed and is more closely related to proliferation and growth, while Akt2 has been reported to be the most predominant form in insulin-sensitive tissues that controls energy metabolism [22,23,24,25].

**Figure 5:**
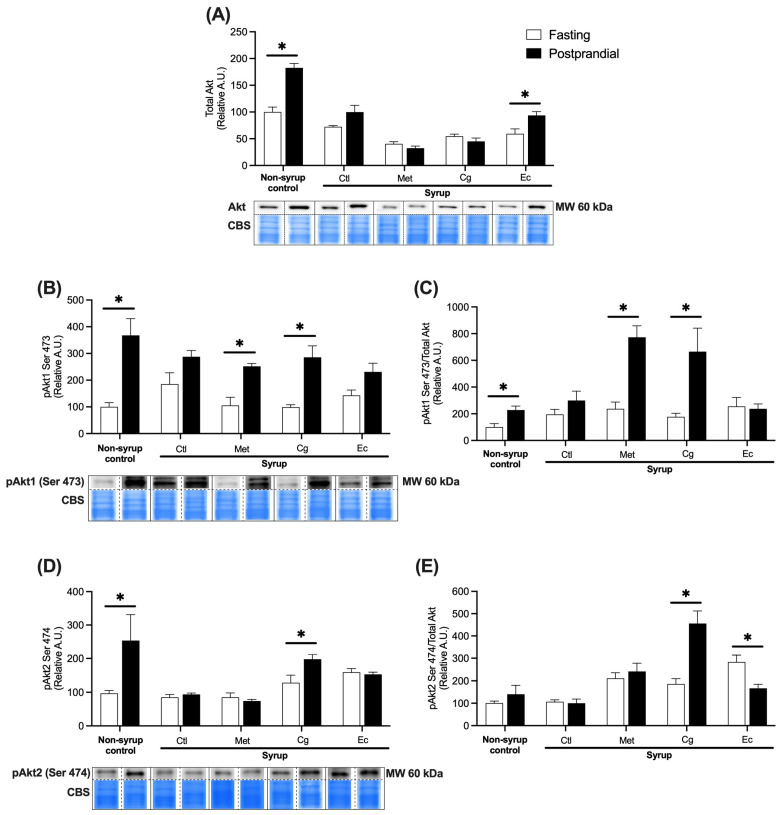
Relative abundances of total Akt and Akt phosphorylated at Ser 473 (pAkt1) and Ser 474 (pAkt2) in liver under fasting and postprandial conditions normalized to total protein: (**A**) Relative abundance of total Akt. (**B**) Relative abundance of pAkt1. (**C**) Relative abundance of pAkt1/total Akt. (**D**) Relative abundance of pAkt2. (**E**) Relative abundance of pAkt2/total Akt. Data are expressed as mean ± SEM (*n* = 4 per condition). Unpaired *t*-tests were performed to compare the fasting (white bars) with postprandial (black bars) conditions of each group; * indicates significant difference between conditions, *p* < 0.05. Total loaded protein was visualized with Coomassie blue staining (CBS) and representative blots are shown below the graphs. CBS was used to normalize the abundance of Akt isoforms relative to the total protein load per lane. The dotted lines in the blots represent the separation of samples shown on the same membrane. Ctl, syrup control; Met, syrup group treated with metformin (850 mg/kg b.w.); Cg, syrup group treated with *C. guatemalensis* extract (30 mg/kg b.w.); Ec, syrup group treated with *E. cymosum* extract (470 mg/kg b.w.).

In liver, postprandial stimulation led to a 1.82-fold increase in total Akt abundance in non-syrup control rats (*p* = 0.0016) (Figure 5A). This response was mitigated by syrup consumption and significantly recovered by Ec, whose treatment augmented the Akt abundance 1.57 times in the postprandial state (*p* = 0.0334). Concerning the activation of both Akt isoforms in the non-syrup control, the glucose load promoted the phosphorylation of Akt1 by 3.67 times (*p* = 0.0056) and of Akt2 by 2.53 times (*p* = 0.0196) (Figure 5B,D), indicating functional insulin signaling. On the other hand, syrup treatment markedly inhibited this response by decreasing the difference between fasting and postprandial levels of both phosphorylated isoforms. Met and Cg treatments reversed this phenomenon by promoting pAkt1 upregulation by 2.39 (*p* = 0.0195) and 2.9 times (*p* = 0.0008), respectively, while only Cg increased Akt2 phosphorylation by 1.53 times (*p* = 0.0377). When analyzing the ratio between the amount of total Akt and the phosphorylated forms of Akt1 and Akt2 (Figure 5C,E), treatments with Met and Cg resulted in a 3.27- (*p* = 0.0030) and 3.77-fold increase (*p* = 0.0062) in Akt1 phosphorylation, whereas Cg increased pAkt2 by 2.45 fold (*p* = 0.0045). Interestingly, Ec treatment reduced pAkt2 by 0.58 fold in the postprandial phase (*p* = 0.0144), suggesting a worse response to insulin. These results indicate that Cg, but not Ec, reverses the damage on hepatic insulin signaling caused by chronic syrup ingestion.

**Figure 6:**
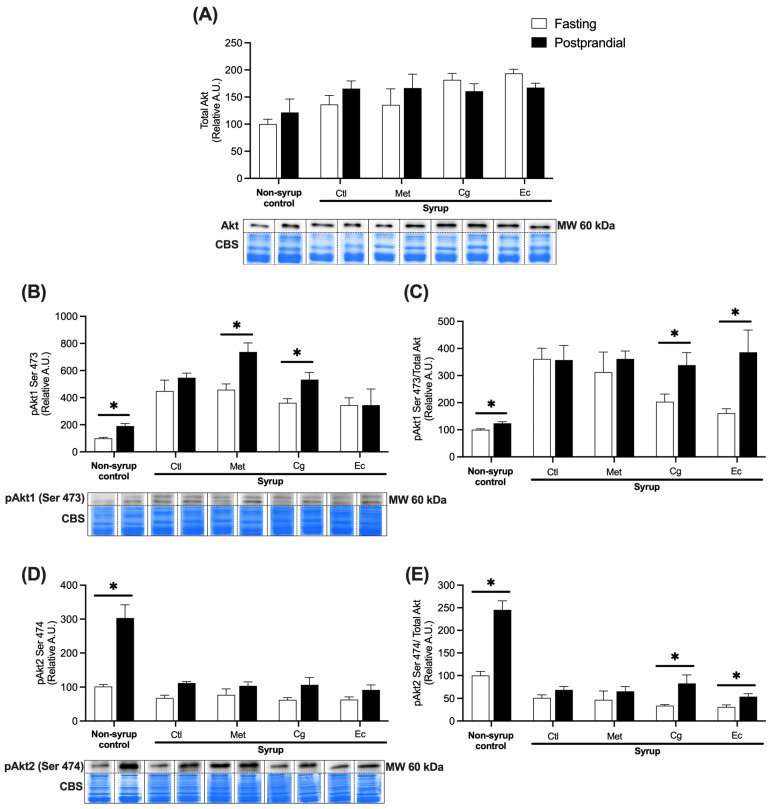
Relative abundances of total Akt and Akt phosphorylated at Ser 473 (pAkt1) and Ser 474 (pAkt2) in gastrocnemius muscle under fasting and postprandial conditions normalized to total protein: (**A**) Relative abundance of total Akt. (**B**) Relative abundance of pAkt1. (**C**) Relative abundance of pAkt1/total Akt. (**D**) Relative abundance of pAkt2. (**E**) Relative abundance of pAkt2/total Akt. Data are expressed as mean ± SEM (*n* = 4 per condition). Unpaired *t*-tests were performed to compare the fasting (white bars) and postprandial (black bars) conditions of each group; * indicates significant difference between conditions, *p* < 0.05. Total loaded protein visualized with Coomassie blue staining (CBS) and representative blots are shown below the graphs. CBS was used to normalize the abundance of Akt isoforms relative to the total protein load per lane. The dotted lines in the blots represent the separation of samples shown on the same membrane. Ctl, syrup control; Met, syrup group treated with metformin (850 mg/kg b.w.); Cg, syrup group treated with *C. guatemalensis* extract (30 mg/kg b.w.); Ec, syrup group treated with *E. cymosum* extract (470 mg/kg b.w.).

In gastrocnemius muscle, no significant changes were observed with any of the treatments in terms of the amount of total Akt (Figure 6A). Regarding non-syrup control, postprandial stimulation resulted in a significant 1.9-fold increase in pAkt1 (*p* = 0.0057) and a 3-fold increase in pAkt2 (*p* = 0.0006) relative to their fasting baseline levels (Figure 6B,D). Syrup consumption damaged this response by inhibiting the postprandial phosphorylation of both isoforms. Nevertheless, treatments with Met and Cg increased pAkt1 levels in postprandial conditions by 1.6 (*p* = 0.0205) and 1.5 times (*p* = 0.0233), respectively (Figure 6B). Contrastingly, no treatment restored the pAkt2 isoform response (Figure 6D). When analyzing the ratio between the amount of both phosphorylated isoforms to total Akt, both Cg and Ec treatments significantly increased the postprandial levels of pAkt1 (*p* = 0.0452; *p* = 0.0267) and pAkt2 (*p* = 0.0096; *p* = 0.0251) relative to the amount of total Akt, suggesting that both plant extracts restored the proportion of activated insulin signaling pathways in this tissue (Figure 6C,E).

Taken together, these results indicate that Cg and Ec extracts improve insulin function in a tissue-specific manner: Cg can restore insulin sensitivity in the liver and muscle, while Ec only has a positive effect on muscle.

### 2.5. Pancreatic β-Cell Mass and Insulin Levels

To determine whether syrup consumption or treatments modified the population of pancreatic β cells or the amount of insulin within the islets, the insulin-positive area and insulin intensity relative to islet area of the different groups were quantified under fasting and postprandial conditions.

**Figure 7:**
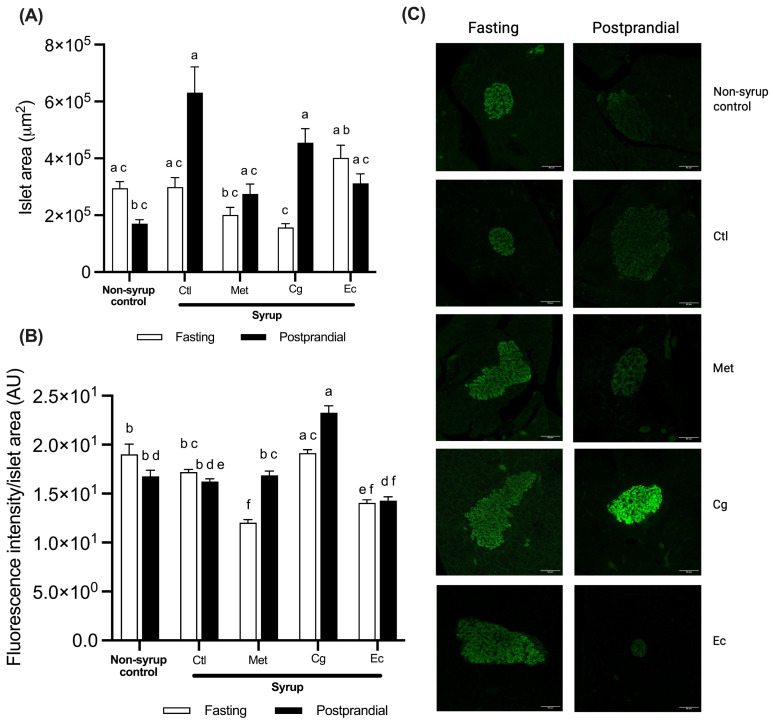
Insulin abundance in pancreatic islets: (**A**) Insulin-positive area in the pancreatic islets. (**B**) Insulin levels in the pancreatic islets. (**C**) Representative confocal micrographs of pancreatic islets. Scale bar, 90 μm. Data are expressed as mean ± SEM (*n* = 3 per condition). One-way ANOVA followed by Tukey post hoc tests were applied to compare the means among groups. Different letters over the bars indicate statistically significant differences (a > b > c > d > e > f), *p* < 0.05. Ctl, syrup control; Met, syrup group treated with metformin (850 mg/kg b.w.); Cg, syrup group treated with *C. guatemalensis* extract (30 mg/kg b.w.); Ec, syrup group treated with *E. cymosum* extract (470 mg/kg b.w.).

In the non-syrup control group, there was a non-significant 42% reduction in the postprandial insulin-positive area compared to fasting, while syrup consumption showed a non-significant increase in the postprandial insulin-positive area by 111% in relation to the fasting condition (Figure 7A). Interestingly, although Cg treatment tented to stimulate the most evident decrease in the insulin-positive area during fasting among all treatments, it significantly augmented the area in the postprandial state by 190% compared with its own fasting condition (*p* = 0.0006).

When analyzing the insulin levels, non-syrup rats showed similar abundance in both fasting and postprandial states (Figure 7B). However, in the fasting condition, a subpopulation of islets with a higher amount of insulin was observed. In contrast, although no significant differences in insulin levels were detected between fasting and postprandial states in rats treated with syrup, treatment with syrup led to the loss of the islet subpopulation with the highest insulin levels during fasting. This may suggest that rats treated with syrup have a lower amount of insulin to secrete in response to glucose stimulation in fasting condition. In fact, both postprandial and fasting insulin levels measured in the serum of syrup rats during the OGTT was similar to those observed in the postprandial period of control rats without syrup (Figure 4F). It is noteworthy that, although the same postprandial islet insulin levels were detected in both groups, the levels observed in the syrup group were distributed over a larger area within the islet compared to the insulin-positive area of the islet in the healthy control group (Figure 7A). This evidence suggests that treatment with syrup may modify islet architecture affecting the β-cell population and impairing the postprandial insulin secretory response.

Syrup-treated rats with Met had significantly less islet insulin levels during fasting than postprandial rats (*p* < 0.0001) (Figure 7B). Moreover, they had significantly lower insulin levels during fasting than negative control rats treated with syrup under the same conditions (*p* < 0.0001). This behavior could have been due to the insulin-sensitizing effect of Met, which indirectly promoted a decrease in pancreatic functional overload by improving fasting glucose regulation (Figure 3). In this context, the islet could be responding with a decrease in insulin-fasting insulin synthesis and secretion and with the restoration of GSIS in the postprandial state. Cg-treated rats, on the other hand, exhibited markedly increased insulin levels under postprandial condition, being the group and condition with the highest insulin levels. Furthermore, a subpopulation of islets with higher insulin levels was observed in the postprandial state (Figure 7B). These results suggest that treatment with Cg may be increasing pancreatic β-cell function through promoting insulin synthesis and positive regulation of the insulin secretory machinery. Finally, the Ec-treated rats had the same amount of insulin within the islet in the fasting and postprandial state. These values were similar to those of the Met group in fasting (Figure 7B). This suggests that Ec consumption could be increasing the insulin secretory response, causing the islet to have a lower quantity of insulin within the islet than in the bloodstream, both in fasting and postprandial states (Figure 4F).

By integrating the results, it could be concluded that chronic consumption of syrup modifies pancreatic islet population and impairs the insulin secretory response, probably due to functional overload. Plant extracts, unlike Met, appear to exert a direct impact on pancreatic islets, either by increasing insulin synthesis, exerting a positive effect on the insulin secretion machinery, or restoring pancreatic islet population.

## 3. Discussion

Traditional medicine, in addition to representing the cultural and biological wealth of a specific population, is a promising resource available for the search for new molecules with therapeutic effects. The adoption of new species through a “trial and error” approach for the treatment of a particular disease allows for the enrichment of medicinal species in the traditional medicine system. This is advantageous when analyzing molecules or mixtures of molecules for a specific disease since biological richness is linked to chemical structural richness that could be correlated with a specific pharmacological effect. In the context of metabolic diseases, mixtures of molecules could have a better therapeutic effect due to the multiple pathophysiological abnormalities that occur [26].

**Figure 8:**
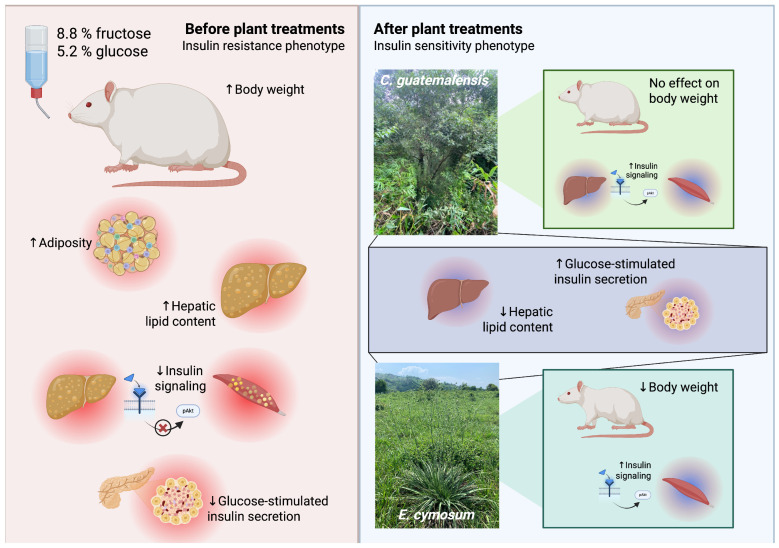
Summary of the effects of *C. guatemalensis* and *E. cymosum* on the IR phenotype. Chronic consumption of a syrup containing 8.8% fructose and 5.2% glucose increased body weight, adiposity, and hepatic lipid content, in addition to impairing the postprandial insulin response in liver and muscle and the glucose-stimulated insulin secretion response. Treatment with plant extracts differentially reversed some of these conditions. Specifically, *C. guatemalensis* improved insulin signaling in the liver and muscle, while *E. cymosum* did so only in muscle. Created with BioRender.com.

Patients with T2D often turn to medicinal plants as adjuvants when the disease is in its advanced stages. However, at this phase, hyperglycemia is already present, so IR coexists in a cellular environment where pancreatic β cells are already dysfunctional. Thus, current therapies involve early positive lifestyle changes and/or pharmacological intervention with the aim of preventing the progression of the “prediabetic” condition to overt diabetes [9]. In this sense, our research demonstrated that Cg and Ec, two species traditionally used for the treatment of T2D, are capable of reversing the damage to the insulin pathway response in a tissue-specific manner caused by exposure to syrup and possibly promote improved β-cell function (Figure 8).

Furthermore, it has been reported that most patients with diabetes consume plant infusions for extended periods of about a month, so understanding the toxic properties of plants is essential. Although no apparent toxic effects were registered after the four-week treatment of Cg and Ec, chronic toxicity testing is recommended in future studies. However, acute toxicity tests performed in previous works with the species did not reveal any abnormal physical or behavioral changes [14]. In this regard, some clerodane diterpenoids and phenolic acids may have toxic properties as they have demonstrated cytotoxic effects against cancer cell lines [27,28].

It is well known that chronic consumption of high-fructose syrups promotes deleterious effects on metabolism by increasing lipid content in the liver (hepatic steatosis), which will influence the generation of an IR phenotype due to increased adiposity and the subsequent appearance of a proinflammatory environment [29]. In the long term, hepatic IR causes fasting hyperglycemia because glucose production is not suppressed. In this context, not only are gluconeogenesis and glycogenolysis active, but lipogenesis is also increased. This phenomenon, known as “selective hepatic insulin resistance” or “selective insulin resistance and response”, causes hepatic steatosis. Furthermore, chronic overnutrition activates several lipogenic factors independent of the insulin signaling pathway, such as the substrate-driven redistribution of circulating fatty acids caused by IR in adipose tissue, which contributes a significant amount of triglycerides [30].

In the present investigation, an exacerbated accumulation of triglyceride and cholesterol content in the liver was observed in the syrup-treated rats, which could indicate signs of hepatic steatosis. Plant extracts significantly reduced liver lipid content, showing an effect similar to that reported for Met [31]. Since the treatments did not induce significant changes in VAT weight and/or in b.w., this reducing effect on hepatic lipid content could be explained by a decrease in IR in the adipose tissue itself, which would result in a reduction in lipolysis and, consequently, in a lower uptake of free fatty acids by the liver [32]. It is important to note that the decrease in b.w. promoted by Ec could be due to a reduction in subcutaneous adipose tissue; however, this could not be determined because of study limitations. On the other hand, this effect could also have been due to the high dose of Ec relative to that of Cg. Water has been associated with a higher total yield because of the extraction of a wider range of polar compounds and, therefore, the final extraction yield leads to a relatively higher dosage. In this sense, the objective of the work was to evaluate the effect of the extracts at their traditional doses, so the reduction in b.w. could have been due to the higher traditional dose of Ec.

In addition, the extracts could be modulating lipid accumulation and adipogenesis, as it has been reported that the compounds present in both extracts, like phenolic acids and clerodane diterpenes, exhibited a lipid-lowering effect by activating fatty acid oxidation and an anti-obesogenic effect by inhibiting adipogenesis [33,34]. Moreover, plant extracts were shown to restore the postprandial insulin response through increased phosphorylation of the two main Akt isoforms. Although Akt2 is specifically linked to metabolism control, Akt1 polymorphisms have been associated with the development of T2D [35]. Therefore, to evaluate the impact of the extracts on insulin sensitization, the phosphorylation of both isoforms was used as markers.

It has been reported that an IR phenotype differentially impacts the basal phosphorylation status of both isoforms in addition to impairing the postprandial phosphorylation response to insulin. IR associated with high-fat diet (HFD) exposure in mice causes the hyperphosphorylation of Akt1 in fasting liver and muscle, whereas basal and stimulated Akt2 phosphorylation is decreased in human muscle [36,37,38]. This would explain why treatment with the syrup, which is associated with an IR phenotype, increases the basal phosphorylation state of Akt1 and decreases the basal amount of phosphorylated Akt2. However, although the plant extracts did not reverse this phenomenon in the fasting state, they were able to restore the phosphorylation state under postprandial conditions, indicating an improvement in the response of the insulin signaling pathway. Cg was shown to restore the pAkt response of both isoforms in liver and muscle, which could be correlated with its potent effect in lowering FBG levels and the exacerbated decrease in blood glucose during the OGTT. Epicatechin, a flavonoid present in Cg extract, has been shown to increase pAkt1 in HepG2 cells under non-insulin-resistant conditions, which could explain the potent effect of Cg on the liver [39]. However, the major compounds in the extract are *ent*-clerodane diterpenoids [18], whose effects on insulin sensitivity have not yet been studied. On the other hand, Ec only enhanced the response of both Akt isoforms in muscle, limiting its potential therapeutic effect on IR compared with Cg. Rosmarinic acid, one of the major compounds of Ec, has been shown to restore insulin-stimulated glucose uptake in muscle cells exposed to palmitate, although it has also been documented to exert a negative impact on the Akt signaling pathway, showing a potential inhibitory effect on the proliferation and invasion of hepatocellular carcinoma cells [27,40].

In this context, the analysis of Akt phosphorylation by Western blot positively correlates with previous results regarding glucose regulation in dynamic tests and partially with the surrogate indices obtained. As the liver is directly involved in fasting glucose homeostasis and the muscle is primarily responsible for peripheral glucose regulation in the postprandial state, the potent hypoglycemic effect of Cg in fasting, as well as the improvement of glucose tolerance in OGTT and the potentiation of the insulin effect in IPITT, can be explained by the enhancement of insulin signaling in the liver and muscle, respectively. Furthermore, Ec improves glucose tolerance and exacerbates the hypoglycemic effect of insulin by promoting a greater total glucose removal and increasing the rate of glucose elimination, suggesting a better regulation of insulin signaling in muscle. However, although both extracts also showed the ability to restore GSIS, they were not able to decrease fasting hyperinsulinemia. Therefore, the effect of the extracts on the insulin content in the pancreatic islets in fasting and postprandial state was evaluated next.

Regarding β-cell function, both plant extracts were shown to have a positive impact by restoring GSIS after glucose loading. This effect was similar to that observed by Met; nevertheless, islet immunohistochemistry revealed that both Cg and Ec could be exerting a direct effect on insulin synthesis and secretion, as well as on the islet population. This result is highly relevant to the study, as the insulin-related indices HOMA-IR and QUICKI implied that plants might not have an insulin-sensitizing effect. Therefore, it could be hypothesized that plant extracts may promote greater basal insulin secretion in response to IR due to a direct effect on the β cell, generating higher insulin levels than syrup control, which could cause the downregulation of the insulin receptor and, thus, decreased insulin signaling, as reported previously [41]. In this regard, it is encouraged that studies on IR should be conducted using not only surrogate indices related to insulin, but also other surrogate indices, such as TyG index, and dynamic tests to avoid false negatives, since, as observed in the TyG index and dynamic tests, both Cg and Ec were able to reduce IR, enhance the effect of insulin, and improve glucose tolerance.

By restoring the biphasic kinetics of the GSIS response, both plant extracts also demonstrated the ability to improve pancreatic islet function by promoting mechanisms that enhance insulin synthesis and secretion capacity, as well as promote less degradation, in addition to possibly normalizing pancreatic islet mass and population. Accordingly, islet insulin appears to correlate with the serum insulin levels detected during the OGTT (Figure 4F). Although the levels of fasting serum insulin in the Cg group were slightly lower than those of the syrup control group in the same condition, the amount of fasting islet insulin occurred in a significantly smaller area. In addition, the postprandial serum insulin levels of this group were similar to those of the Met group. On the other hand, fasting and postprandial serum insulin levels of Ec-treated rats can also be correlated with islet insulin levels, since the Ec group showed the highest serum insulin values in both conditions. It is important to consider that the amount of insulin present in the islet of Ec group in fasting is significantly lower than that of the negative syrup control and that, in the postprandial condition, the amount of insulin present in the islet is found in a much smaller area than that of the same group. Thus, Ec treatment could be restoring the pancreatic cell population.

These results are similar to previous studies performed with sitagliptin analogues, where it was shown that the chronic inhibition of the enzyme dipeptidyl peptidase 4 (an incretin enhancer) dose-dependently increased the number of insulin-positive β cells in islets, leading to the normalization of β-cell mass and β-cell-to-α-cell ratio in HFD-STZ-induced diabetic mice, in addition to a rise in islet insulin content and improved GSIS in isolated islets [42]. Phenolic compounds, such as eugenol, have been shown to enhance insulin secretion and content in isolated pancreatic islets [43], while rosmarinic and chlorogenic acids have been reported to preserve β-cell function by inhibiting apoptosis processes [44,45]. However, no effect has been documented for clerodane diterpenes, which opens an area of opportunity for the study of these structural scaffolds on this action mechanism. It is also noteworthy that Cg extract had the greatest effect on islet insulin content, so further studies on this species and its compounds are warranted.

Alternatively, natural products have been demonstrated to enhance insulin sensitivity and secretion through the activation of adenosine 5‘-monophosphate (AMP)-activated protein kinase (AMPK), an intracellular energy sensor that plays a relevant role in controlling whole-body energy homeostasis [46,47]. The activation of this metabolic regulator would result in increased fatty acid oxidation and peripheral glucose uptake, as well as the restoration of GSIS. Previous works suggested that Cg could be an AMPK activator and that Ec could exert an insulin-independent sensitizing effect; therefore, both species may have the capacity of activating this master regulator [14,15]. Moreover, it has been documented that rosmarinic acid, the main compound found in Ec, promotes glucose uptake via AMPK activation and the increase in glucose transporter type 4 (GLUT4) expression [48], while clerodane diterpenes such as tanshinone IIA can inhibit AMPK, indicating that they may possess anticancer properties [49]. In this regard, further studies are needed to evaluate the impact of junceic acid, the most abundant clerodane diterpene in Cg, on AMPK activation.

Current therapies for the treatment of T2D focus on early intervention to prevent the development of hyperglycemia. In this regard, molecules capable of reducing IR and improving pancreatic function are of particular interest for the development of new drugs. Here, it was demonstrated that species used in traditional medicine can improve insulin action in a tissue-specific manner and restore pancreatic function. These results support the potential development of both medicinal plants as future nutraceuticals that can be consumed to prevent the onset of T2D. However, further studies are needed to assess how prolonged use of the extracts would affect pancreatic function, as the continuous synthesis and secretion of insulin may eventually exhaust the β cells.

According to current pharmacological approaches, making β cells healthier is the target goal to delay frank hyperglycemia. Hence, early intervention with molecules or mixtures of molecules capable of exerting a dual effect, improving insulin sensitivity and promoting better functioning of the β cell would result in better glycemic control in patients with T2D. Although both Cg and Ec appear to increase β-cell functionality in the present investigation after four weeks of administration, it is worth noting that they are also sensitizing insulin-dependent tissues to the action of this hormone, so both species are presumably good candidates for longer-term studies. However, before conducting clinical tries, it is necessary to evaluate their potential chronic toxicity, as well as their effect on β-cell survival markers.

## 4. Materials and Methods

### 4.1. Plant Extracts and Phytochemical Composition

Cg was collected in the Department of Chimaltenango, Guatemala. A specimen was deposited at the Deshidrafarmy-Farmaya Herbarium (voucher CFEH 1259). Ec was collected in Huejutla de Reyes, state of Hidalgo, Mexico. A voucher specimen (FC 186813) was deposited at the FC herbarium Mexico.

An ethanol–water extract of Cg bark and an aqueous extract of Ec aerial parts were elaborated based on previous methodologies [14,15,50]. Information regarding plant material collection, voucher numbers, and extract dosages employed in the experiments was sourced from prior studies and determined in accordance with traditional practices. In brief, both doses were calculated following the reasoning that a 70 kg person consumes 20 g of the plant, namely considering traditional usage.

Traditional dose = 20-g yield/70 kg b.w.(1)

Considering traditional dosage and outcomes of previous studies, the doses and type of extract were selected for each species [14,50].

Phytochemical composition of each extract was previously described: junceic acid, crotoguatenoic acid A, crotoguatenoic acid B, formosin F, bartsiifolic acid, rutin, epicatechin, and quercetin were isolated from the ethanol–water extract of Cg bark, while chlorogenic acid, rosmarinic acid, caffeic acid, protocatechuic acid, kaempferol-3-*O*-(2,6-*di-O-trans*-*ρ*-coumaryl)-β-D-glucopyranoside, and quercetin-3-*O*-(2,6-*di-O-trans*-*ρ*-coumaryl)-β-D-glucopyranoside were identified from the aqueous extract of Ec aerial parts [17,18].

### 4.2. Animals

Forty recently weaned male Wistar rats (21 days old) were obtained from the animal facility of the School of Sciences, UNAM. Before starting the study, the rats were acclimatized for two weeks with free access to tap water and Laboratory Rodent Diet 5001 (LabDiet^®^, Richmond, IN, USA; LRD; 28.7 kcal % protein, 57.9 kcal % carbohydrates, and 13.4 kcal % fat; 3.36 kcal/g), maintaining a 12 h light/dark cycle period, 25 °C, and 55% humidity. The procedures performed on the animals were approved by the Academic Ethics and Scientific Responsibility Commission of the School of Sciences, UNAM (approved protocols: T_2019_09_005 and PI_2021_08_23), and carried out according to the Guide for the Care and Use of Laboratory Animals [51].

### 4.3. Experimental Design

**Figure 9:**
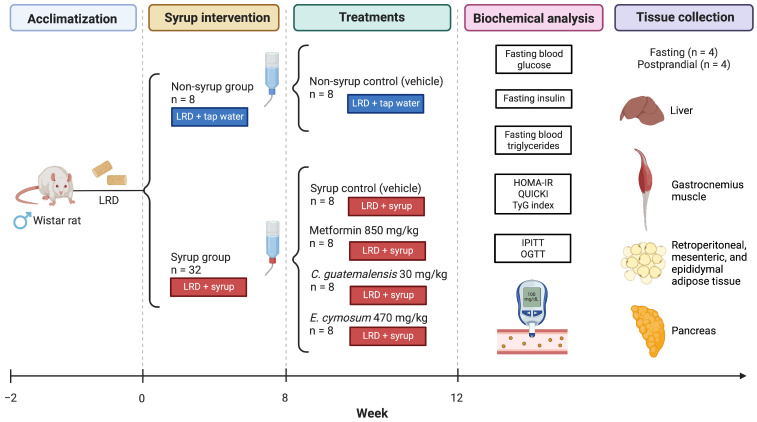
Experimental design. After the eight-week induction period, treatments were daily administered by gavage without removing the syrup stimulus for four weeks. LRD: Laboratory Rodent Diet 5001; HOMA-IR: homeostatic model assessment of insulin resistance; IPITT: intraperitoneal insulin tolerance test; OGTT: oral glucose tolerance test. Created with BioRender.com.

After the two-week acclimatization period, the five-week-old rats continued to be fed the same LRD chow diet but were randomly assigned into two groups based on their drinking solution: one group received tap water (*n* = 8), while the other group received a 10% solution of commercial Madrileña^®^, Mexico City, Mexico, syrup (8.8% glucose and 5.2% fructose; 0.33 kcal/mL) dissolved in tap water [52] (*n* = 32). Eight weeks later, the rats that were previously fed the syrup solution were assigned to four groups (*n* = 8): (1) a negative control that received physiological solution (vehicle); (2) a positive control that were administered metformin (Aurax^®^, Mexico City, Mexico) at 850 mg/kg b.w.; (3) experimental group 1 treated with Cg ethanol–water extract at 30 mg/kg b.w. [50]; and (4) experimental group 2, who received Ec aqueous extract at 470 mg/kg b.w. [14]. All treatments were given by gavage in a single dose at 10 am daily for four weeks and non-syrup rats were administered the vehicle orally during this period (Figure 9). B. w. was monitored weekly, while food and drinking water consumption was recorded every day. FER was calculated by dividing daily b.w. gain (g/day) and food intake (g/day).

At the end of the experiment, rats were fasted overnight to determine FBG, FI, and FBT. FBG and FI values were used to calculate the HOMA-IR and the QUICKI indices, while FBT and FBG were used to determine the TyG index, according to the following formulas (FBG: 1 mmol/L = 18 mg/dL; FI: 1 mU/L = 6 pmol/L) [19,53,54].

HOMA-IR = [FI (mU/L) × FBG (mmol/L)]/22.5(2)

QUICKI = 1/[log FI (mU/L) + log FBG (mg/dL)](3)

TyG index = Ln[FBT (mg/dL) × FBG (mg/dL)/2](4)

Posteriorly, IPITT and OGTT were performed on the animals. Finally, rats were anesthetized to collect tissues in fasting and 30 min after a glucose load (2 g/kg b.w.), simulating postprandial state, in each group (*n* = 4 per condition).

### 4.4. Serum Biochemical Analysis

All blood samples were obtained from the tail vein of the animals. Glucose levels were analyzed with Accu-Chek^®^ (Roche, Indianapolis, IN, USA), active glucometers, while triglyceride levels were quantified with Accutrend^®^ Plus (Roche, Indianapolis, IN, USA). Insulin levels were determined using a specific immunoassay kit (Rat/Mouse Insulin ELISA kit EZRMI-13K EMD Millipore/Merck, Burlington, MA, USA) from plasma obtained by centrifugation of the blood samples at 12,000 rpm for 10 min.

### 4.5. Metabolic Tolerance Tests

IPITT was carried out by first measuring basal blood glucose. Immediately after, regular rapid-acting insulin (Aurax^®^) was injected intraperitoneally at 1 U/kg b.w. and blood glucose was measured every 5 min for 20 min. The results obtained from IPITT were used to calculate the kITT values [55], which were determined by multiplying the linear slope of the natural log glucose values from 5 to 15 min by −100.

On the other hand, OGTT was performed by administering a glucose load (2 g/kg b.w.) after determining basal glycemia. Then, blood glucose was measured every 30 min for two hours. In addition, blood samples were obtained for subsequent quantification of insulin levels at all time points. The insulinogenic index (IGI) was calculated using glucose and insulin values determined at times 0 and 30 during the OGTT as follows [56].

IGI = [I_30min_ − I_0min_ (pmol/L)]/[BG_30min_ − BG_0min_ (mmol/L)] (5)

### 4.6. Tissue Collection

All animals were anesthetized to collect the pancreas, liver, gastrocnemius muscle, and VAT: rVAT, mVAT, and eVAT. After being carefully removed and washed with physiological solution (0.9% NaCl), all tissues except pancreas were immediately weighed, frozen, and stored at −40 °C for later analysis. On the other hand, pancreatic tissue was fixed in a 4% paraformaldehyde PBS solution for further processing.

### 4.7. Determination of Hepatic Lipid Content

Fragments of approximately 100 mg were cut from each liver. Subsequently, 20% homogenates were prepared in buffer 0.25 M sucrose, 1 mM EDTA, 5 mM HEPES, pH 7.4, which were then centrifuged at 1000× *g* for 10 min at 4 °C. The supernatants were recovered and used to quantify triglyceride and total cholesterol levels by enzymatic colorimetric assays using protocols established by Spinreact^®^, Girona, Spain.

### 4.8. Western Blotting

Protein extraction from the liver and gastrocnemius muscle was performed by placing pieces of each tissue in RIPA lysis buffer, which were subsequently homogenized, incubated on ice for 40 min, and centrifuged for 15 min at 13,000 rpm at 4 °C. The recovered supernatants were aliquoted to be stored at −70 °C. Protein concentration was determined using the Bio-Rad DC Protein Assay-modified Lowry method (Bio-Rad, Hercules, CA, USA).

Overall, 40 µg of total protein from muscle samples and 30 µg of total protein from liver samples were exposed to denaturing conditions. Afterwards, samples were loaded onto 12% polyacridamide gels and separated by SDS-PAGE using a molecular weight marker (BioRad) as a guide. Protein transfer was performed overnight at 4 °C to PVDF membranes, confirmed by Ponceau staining (Sigma Missouri, St. Louis, MO, USA). Membranes were then blocked with 5% low-fat powdered milk for Western blotting (Bio-Rad) for one hour at 26 °C and incubated overnight at 4 °C with primary antibodies anti-Akt cat. 9272, anti-pAkt1 (Ser473) cat. 9018, and anti-pAkt2 (Ser474) cat. 8599 (Cell Signaling Technology, Danvers, MA, USA). Dilutions for liver protein: anti-Akt 1:300, anti-pAkt1 1:300, and anti-pAkt2 1:2000; dilutions for muscle protein: anti-Akt 1:1000, anti-pAkt1 1:500, and anti-pAkt2 1:2000. Then, incubation with anti-rabbit secondary antibody (cat. GtxRB-003-DHRPX, ImmunoReagents, Raleigh, NC, USA) was carried out for 90 min at 26 °C. Protein detection was performed by chemiluminescence with the ECL reagent (Merck Millipore, Burlington, MA, USA) in the ChemiDoc analyzer (Bio-Rad). Finally, the densitometric analysis from the images obtained by the ChemiDoc was performed in the Bio-Rad Image Lab software version 6, and the values were normalized in relation to densitometric values of the total protein content load in each lane obtained by stanning the blots with blue Coomassie (Thermo Fisher Scientific, Waltham, MA, USA). Uncropped Western blot images can be found in the Supplementary Materials.

### 4.9. Islet Immunohistochemistry and Quantification of Fluorescence and Insulin-Positive Area

After fixation, the pancreatic tissue was processed for paraffin embedding; 5 μm sections were obtained and placed on glass slides. Then, the pancreatic tissue sections were deparaffinized in xylol, hydrated with gradual ethane washes (100%, 96%, 70%, and 50%), and, finally, washed with PBS. Subsequently, a blocking perforating solution containing 2% NGS and 0.3% Triton-X in PBS was added for one hour at room temperature. A rabbit polyclonal anti-insulin antibody (1:1000, Polyclonal Antibody to Insulin INS, host: Rabbit, Cloud Clone Corp, Katy, TX, USA) was added and the sample was incubated overnight at 4 °C. Afterwards, secondary antibody FITC-coupled goat anti-rabbit IgG antibody was added (1:200 Aviva Systems, San Diego, CA, USA). DAPI (sc-3598, CAS 28718-90-3, Santa Cruz Biotechnology, Santa Cruz, CA, USA) was used to identify cell nuclei. Mounting was made in MOWIOL solution and coverslipped.

Visualization and microphotography of pancreatic islets were acquired with a Nikon Eclipse 400-2 epifluorescence microscope (Tokyo, Japan). A 488 nm laser was used to detect a 552 nm emission range for FITC, and a 360 nm laser was used to detect a 460 nm emission range for DAPI, using a 20× objective. Background-corrected fluorescence levels were calculated for each sample and the negative controls. Analysis of the specific fluorescence levels of insulin, as well as the insulin-positive area, was carried out using FIJI software (Image J 1.36; Wayne Rasband; National Institutes of Health, Bethesda, MD, USA). Three pancreatic sections were analyzed for each pancreas (*n* = 3 per group), and at least seven islets were analyzed for each section.

### 4.10. Statistical Analysis

All analyzes and graphs were made in GraphPad Prism version 10 (GraphPad Software, San Diego, CA, USA, www.graphpad.com). First, data were assessed for normality and subsequently log-transformed as necessary. One-way ANOVA followed by Tukey post hoc tests were applied to compare means among groups. Unpaired *t*-tests were performed to compare the means of two groups. Non-parametric tests (Kruskal–Wallis followed by Dunn’s tests and Mann–Whitney U tests) were applied if normality was not obtained, even after log-transformation. Statistical differences were considered at *p* < 0.05.

## 5. Conclusions

In summary, the current research demonstrated that the chronic administration of Cg and Ec, two plants traditionally used in the treatment of diabetes, improve glucose homeostasis, particularly increasing glucose and insulin tolerance, decreasing IR, and optimizing insulin secretion. The enhanced glucose metabolism observed after Cg and Ec administration in rats exposed to glucose and fructose in drinking water strongly implies that this effect is mediated by a lower hepatic lipid accumulation without promoting significant changes in visceral adiposity weight and the restoration of β-cell function through changes in islet mass and insulin content, as well as improved insulin action on liver and/or skeletal muscle. Cg reestablishes the postprandial insulin response in both liver and muscle, whereas Ec is only able to improve the response in muscle. These results indicate that early intervention with the treatment of both species could delay the development of T2D. Subsequent studies are recommended to support the findings reported in the present study, in addition to characterizing the effect of the phytochemical components of Cg, whose therapeutic impact turned out to be more remarkable than Ec.

## Data Availability

Data are contained within the article.

## Supplementary Materials

The following supporting information can be downloaded at: https://www.mdpi.com/article/10.3390/ph18101433/s1, Figure S1: Liver Blot Akt chemiluminescence MW 62 kDa anti-Akt 1:300; Figure S2: Liver Blot Akt commasie; Figure S3: Representative samples Akt liver MW 62 kDa anti-Akt 1:300; Figure S4: Liver Blot pAkt1 (Ser 473) chemiluminescence MW 62 kDa anti-pAkt 1:300; Figure S5: Liver Blot pAkt1 (Ser 473) commasie; Figure S6: Liver Representative samples pAkt 1 (Ser 473) MW 62 kDa anti-pAkt1 1:300; Figure S7: Liver Blot pAkt2 (Ser 474) chemiluminescence MW 62 kDa anti-pAkt2 1:2000; Figure S8: Liver Blot pAkt2 (Ser 474) commasie; Figure S9: Liver Representative samples pAkt 2 (Ser 474) MW 62 kDa anti-pAkt2 1:2000; Figure S10: Muscle Blot Akt chemiluminescence MW 62 kDa anti-Akt 1:1000; Figure S11: Muscle Blot Akt commasie; Figure S12: Representative samples Akt muscle MW 62 kDa anti-Akt 1:1000; Figure S13: Muscle Blot pAkt1 (Ser 473) chemiluminescence MW 62 kDa anti-pAkt 1:500; Figure S14: Muscle Blot pAkt1 (Ser 473) commasie; Figure S15: Muscle Representative samples pAkt 1 (Ser 473) MW 62 kDa anti-pAkt1 1:500; Figure S16: Muscle Blot pAkt2 (Ser 474) chemiluminescence MW 62 kDa anti-pAkt2 1:2000; Figure S17: Muscle Blot pAkt2 (Ser 474) commasie; Figure S18: Muscle Representative samples pAkt 2 (Ser 474) MW 62 kDa anti-pAkt2 1:2000.

## Abbreviations

The following abbreviations are used in this manuscript:

Akt	Protein kinase B
AMP	Adenosine 5′-monophosphate
AMPK	(AMP)-activated protein kinase
AUC	Area under the curve
b.w.	Body weight
CBS	Coomassie blue staining
Cg	*Croton guatemalensis*
Ec	*Eryngium cymosum*
eVAT	Epididymal white adipose tissue
FBG	Fasting blood glucose
FBT	Fasting blood triglycerides
FER	Food efficiency ratio
FI	Fasting insulin
GLUT4	Glucose transporter type 4
GSIS	Glucose-stimulated insulin secretion
HFD	High-fat diet
HOMA-IR	Homeostatic model assessment of insulin resistance
IGI	Insulinogenic index
IPITT	Intraperitoneal insulin tolerance test
IR	Insulin resistance
kITT	Rate of glucose disappearance constant
LRD	Laboratory rodent diet 5001
Met	Metformin
mVAT	Mesenteric white adipose tissue
OGTT	Oral glucose tolerance test
QUICKI	Quantitative insulin sensitivity check index
rVAT	Retroperitoneal white adipose tissue
STZ	Streptozotocin
T2D	Type 2 diabetes
TyG	Triglyceride/glucose index
VAT	Visceral white adipose tissue
