# Supplementary material for: Tissue-Specific Enhancement of Insulin Function and Restoration of Glucose-Stimulated Insulin Secretion by Croton guatemalensis Lotsy and Eryngium cymosum F. Delaroche
Source: Pharmaceuticals (Basel). 2025 Sep 24;18(10):1433. doi: 10.3390/ph18101433 (PMC12567235; doi:10.3390/ph18101433)

Figure S1. Liver Blot Akt chemiluminescence MW 62kDa anti-Akt 1:300.

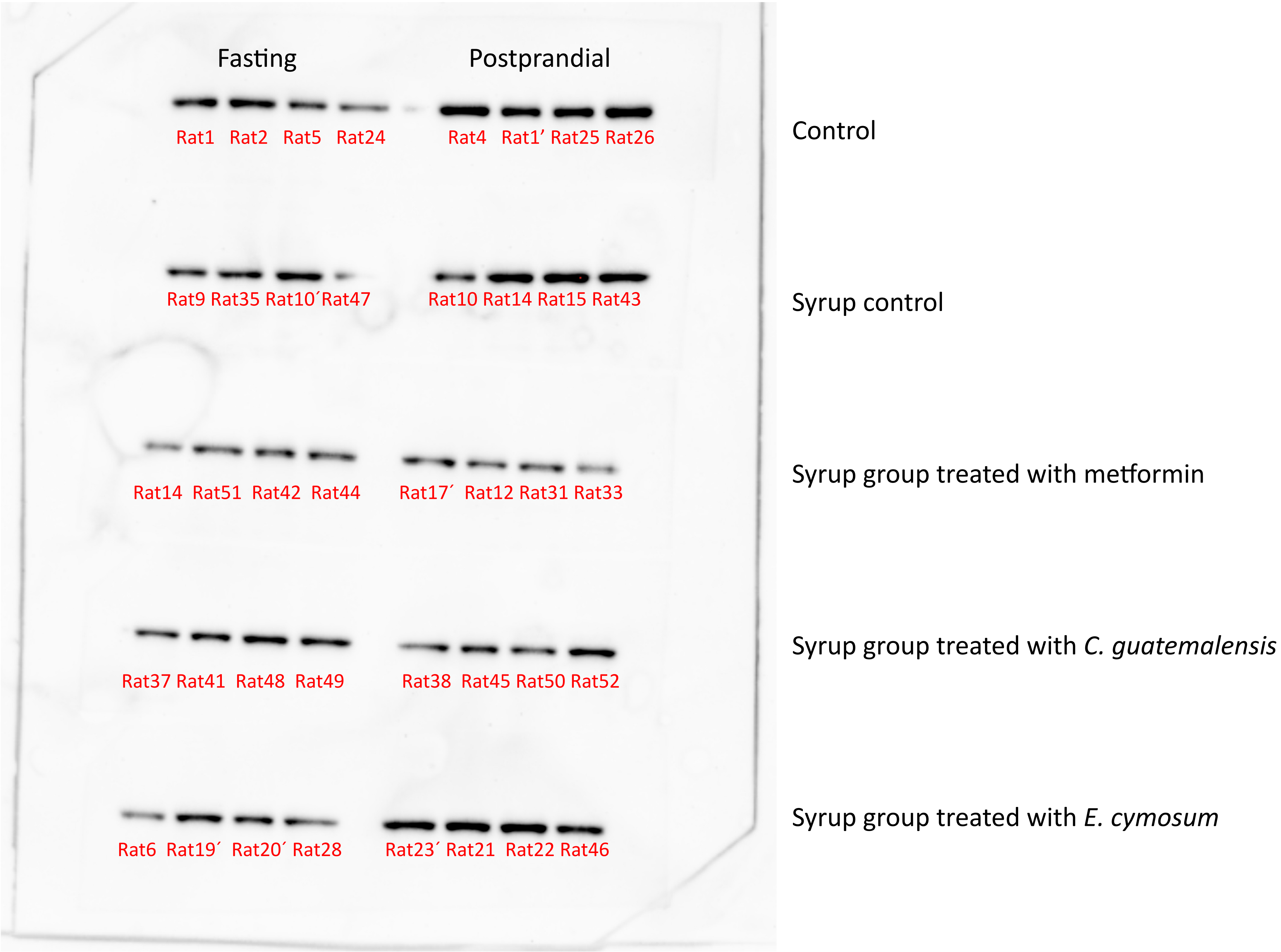

Figure S2. Liver Blot Akt commasie.

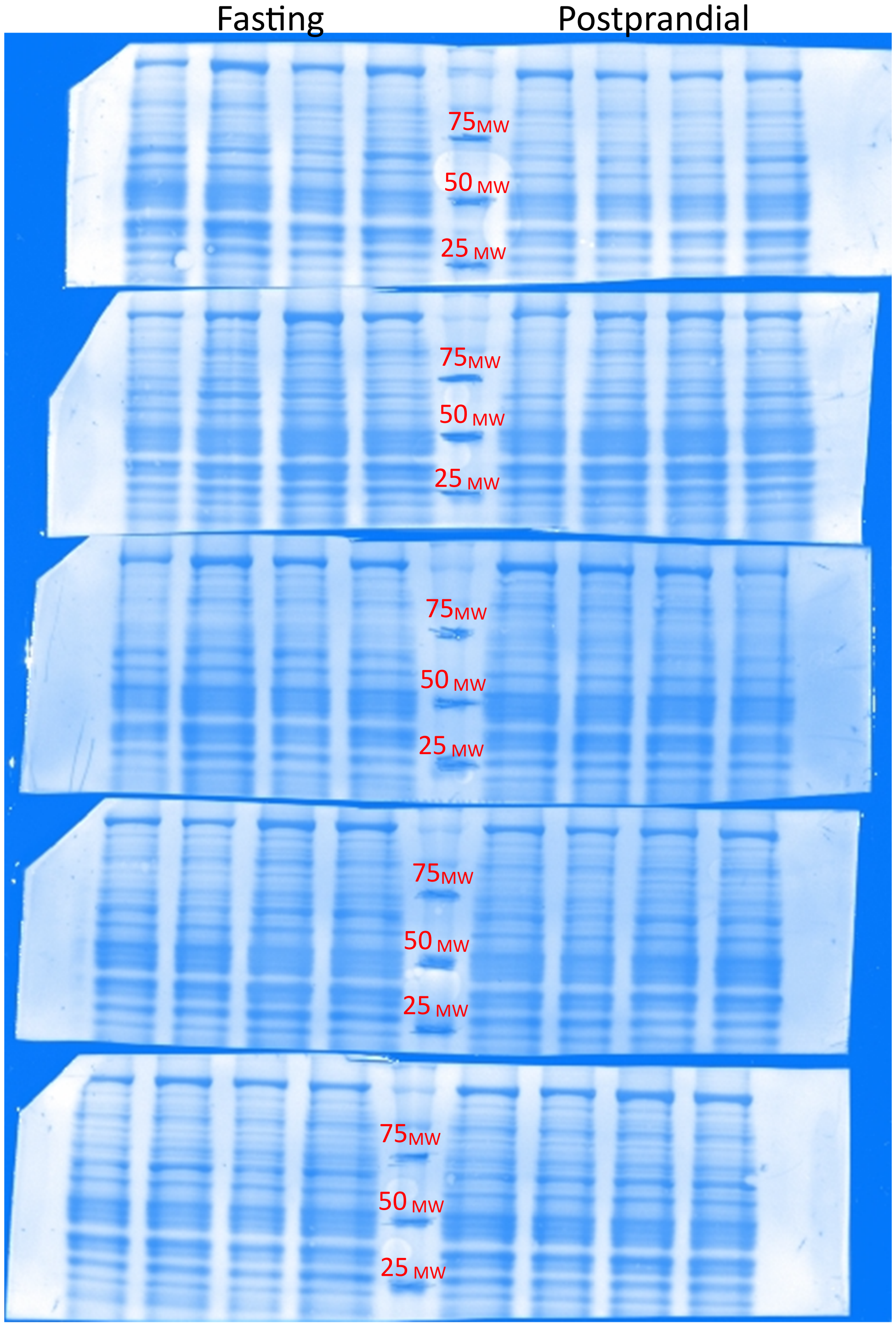

Control

Syrup control

Syrup group treated with metformin

Syrup group treated with *C. guatemalensis*

Syrup group treated with *E. cymosum*

Figure S3. Representative samples Akt liver MW 62kDa anti-Akt 1:300.

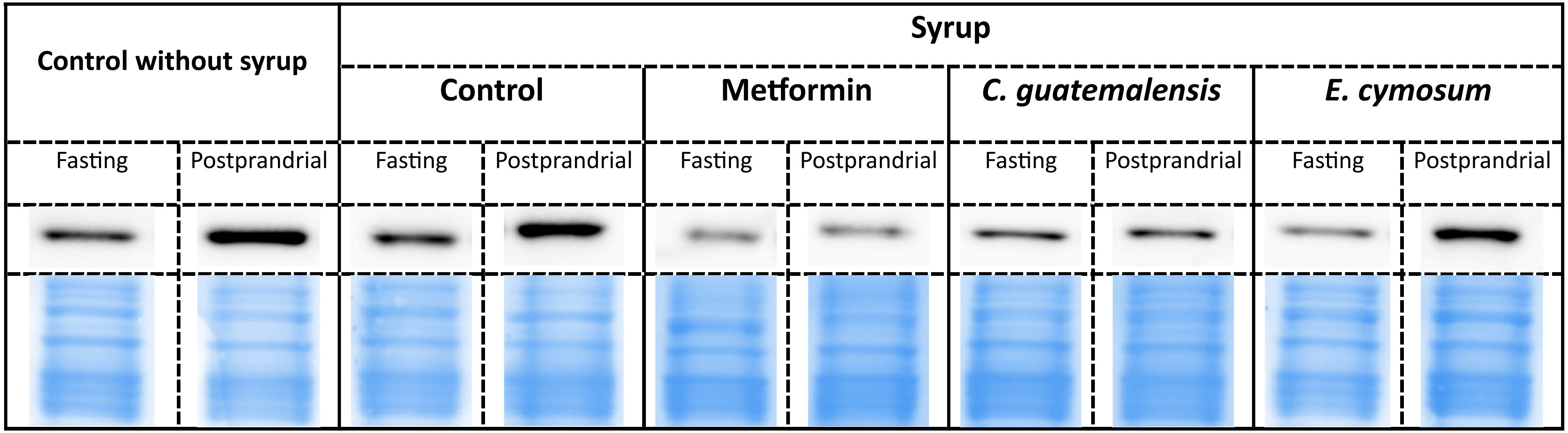

Figure S4. Liver Blot pAkt1 (Ser 473) chemiluminescence MW 62kDa anti-pAkt 1:300.

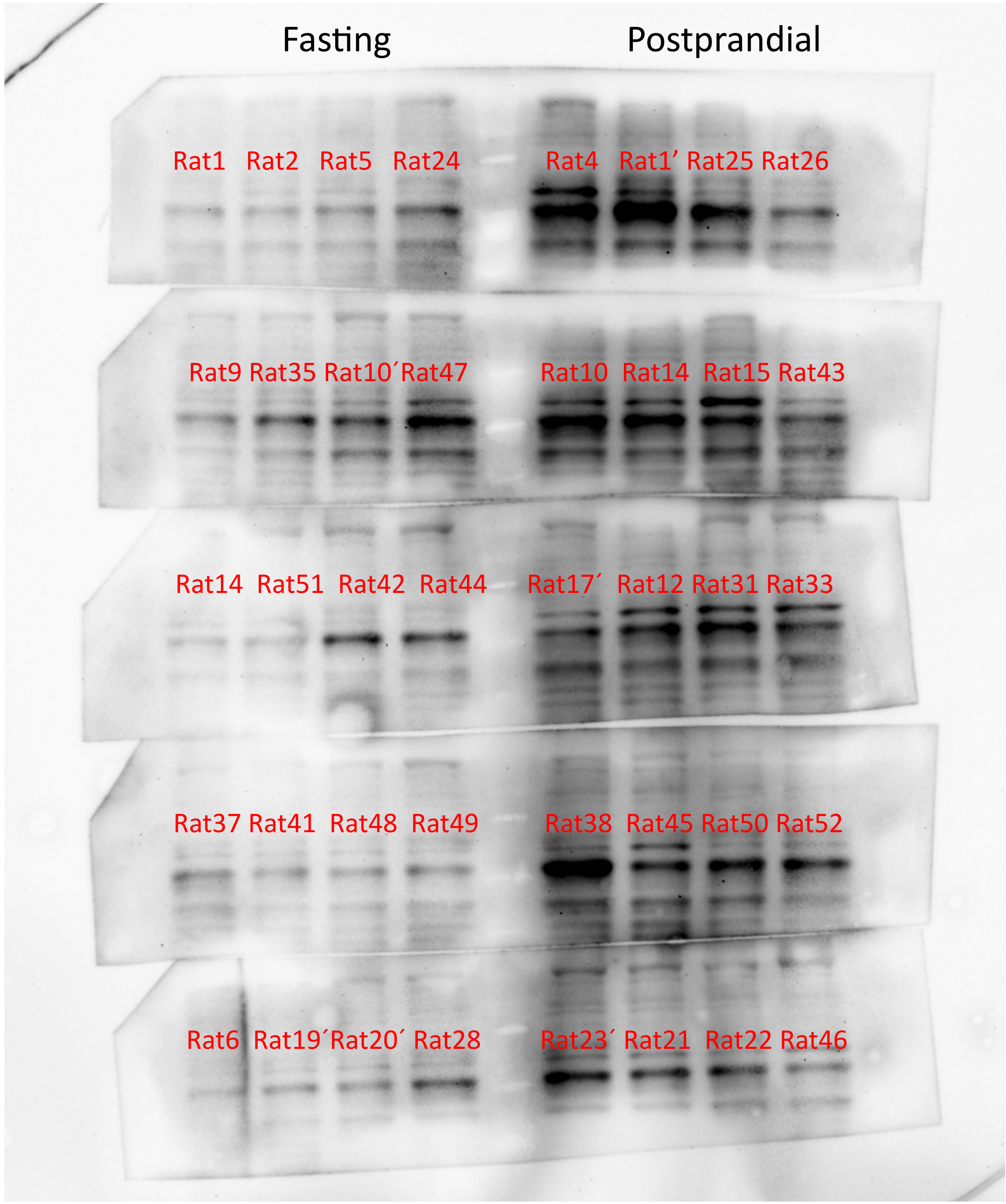

Control

Syrup control

Syrup group treated with metformin

Syrup group treated with *C. guatemalensis*

Syrup group treated with *E. cymosum*

Figure S5. Liver Blot pAkt1 (Ser 473) commasie.

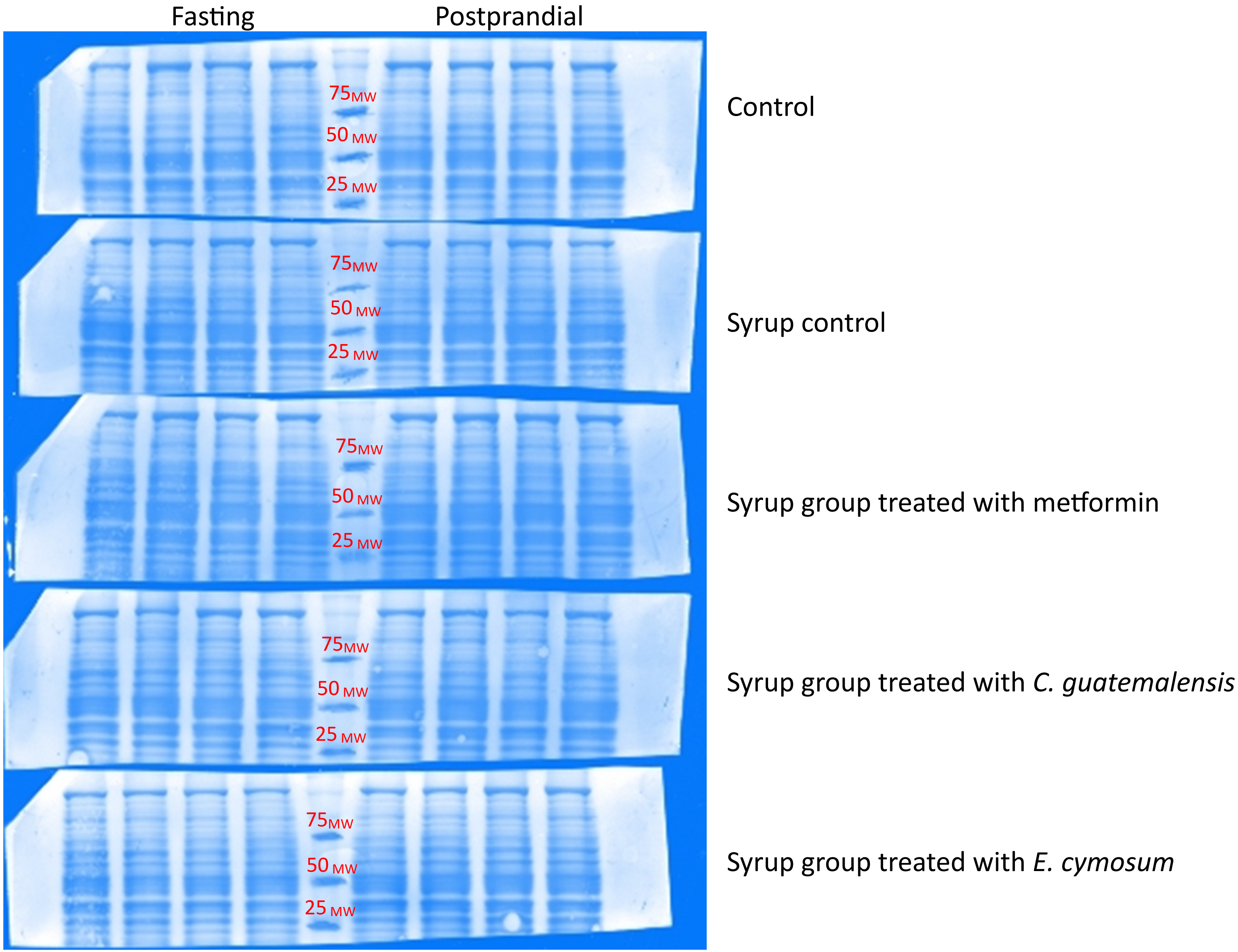

Figure S6. Liver Representative samples pAkt 1 (Ser 473) MW 62kDa anti-pAkt1 1:300.

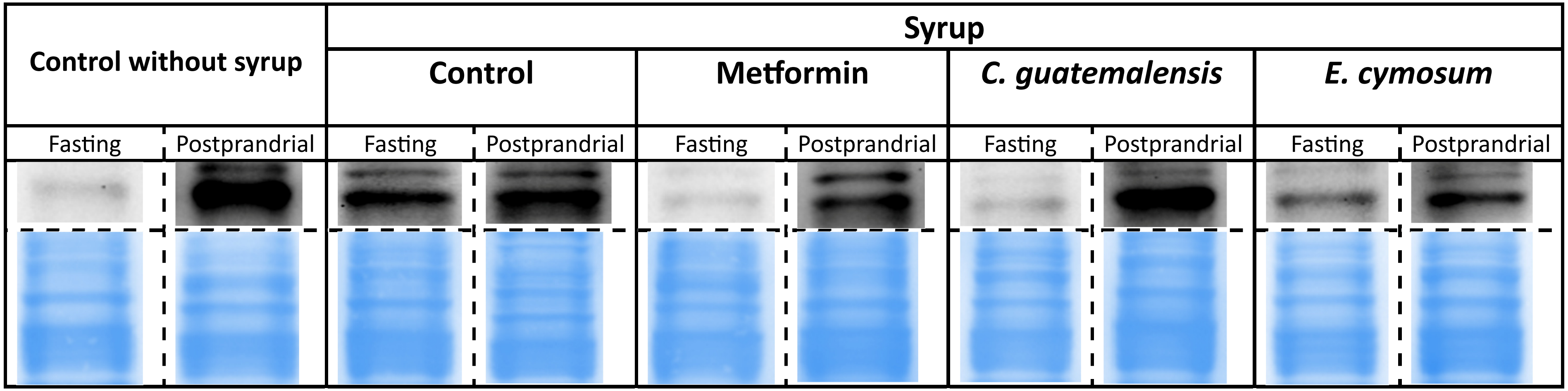

Figure S7. Liver Blot pAkt2 (Ser 474) chemiluminescence MW 62kDa anti-pAkt2 1:2000.

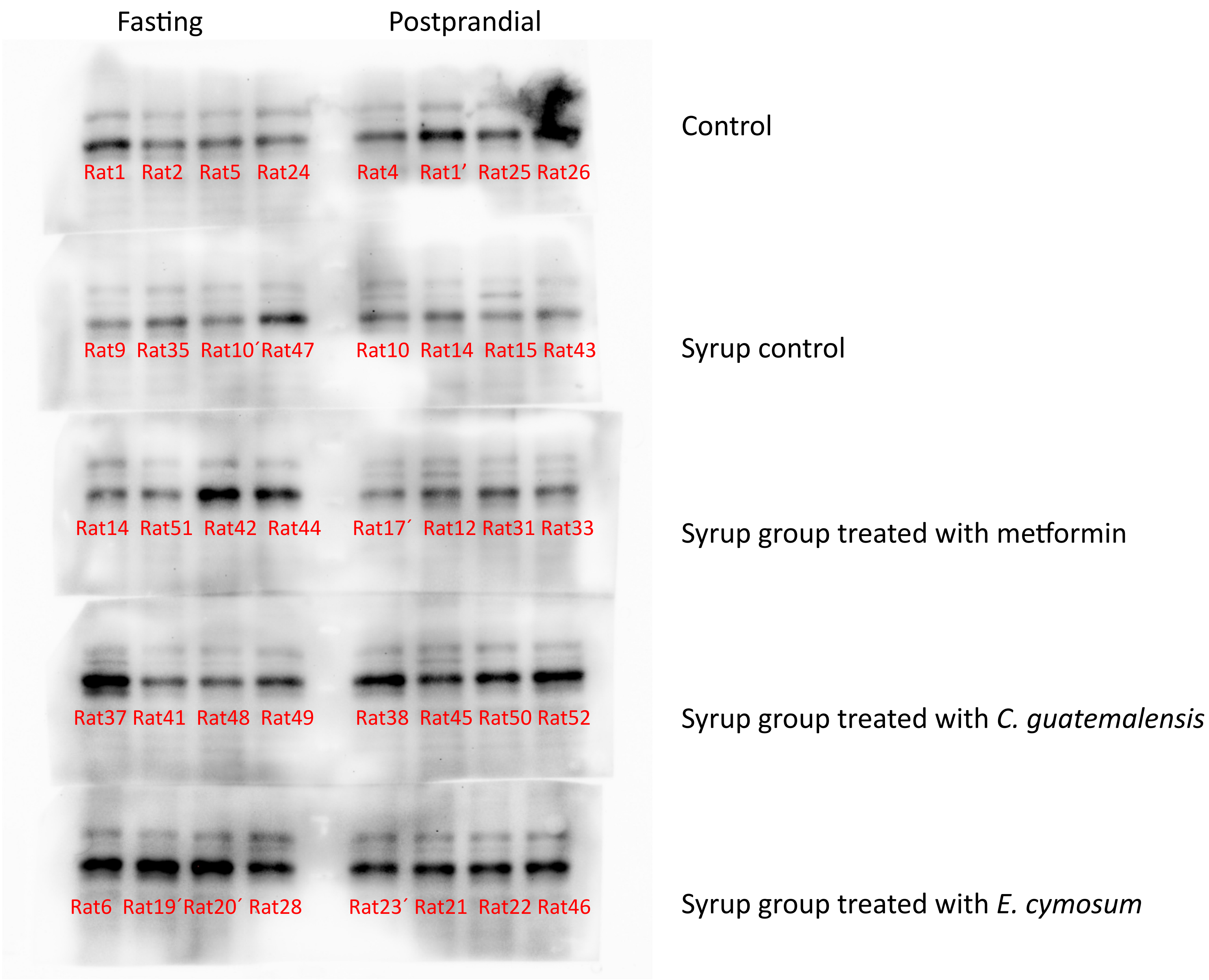

Figure S8. Liver Blot pAkt2 (Ser 474) commasie.

Fasting Postprandial

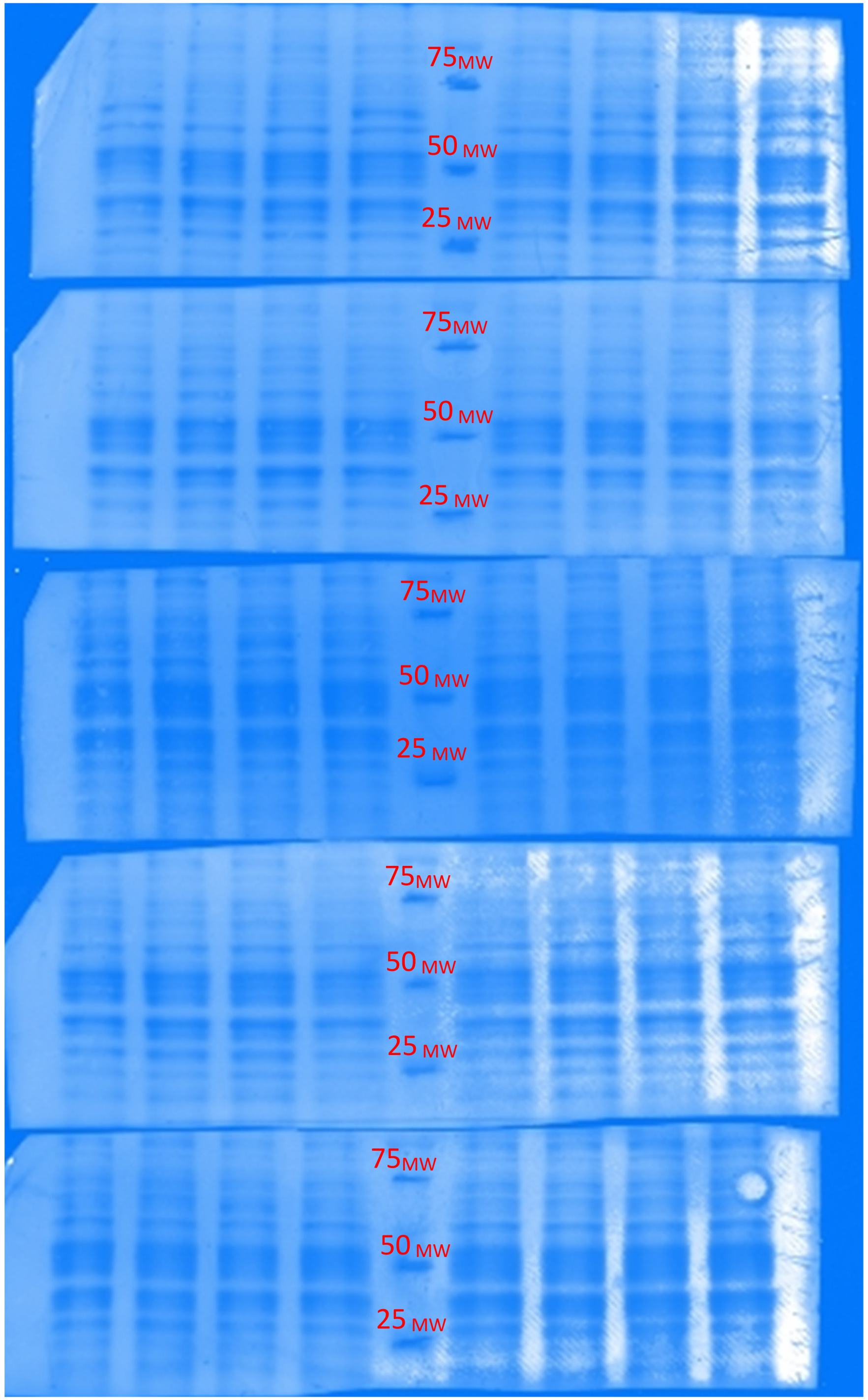

Control

Syrup control

Syrup group treated with metformin

Syrup group treated with *C. guatemalensis*

Syrup group treated with *E. cymosum*

Figure S9. Liver Representative samples pAkt 2 (Ser 474) MW 62kDa anti-pAkt2 1:2000.

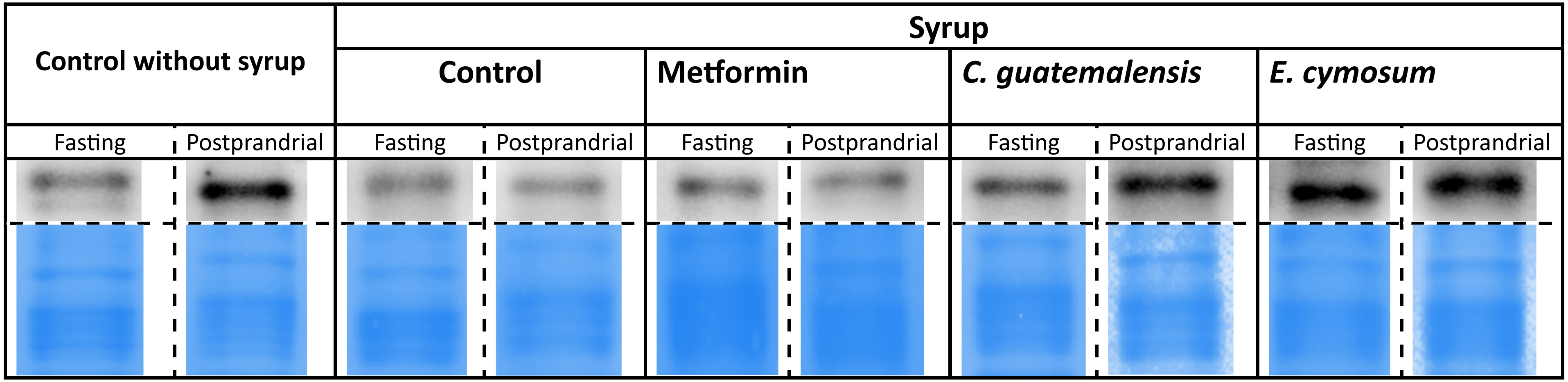

Figure S10. Muscle Blot Akt chemiluminescence MW 62kDa anti-Akt 1:1000.

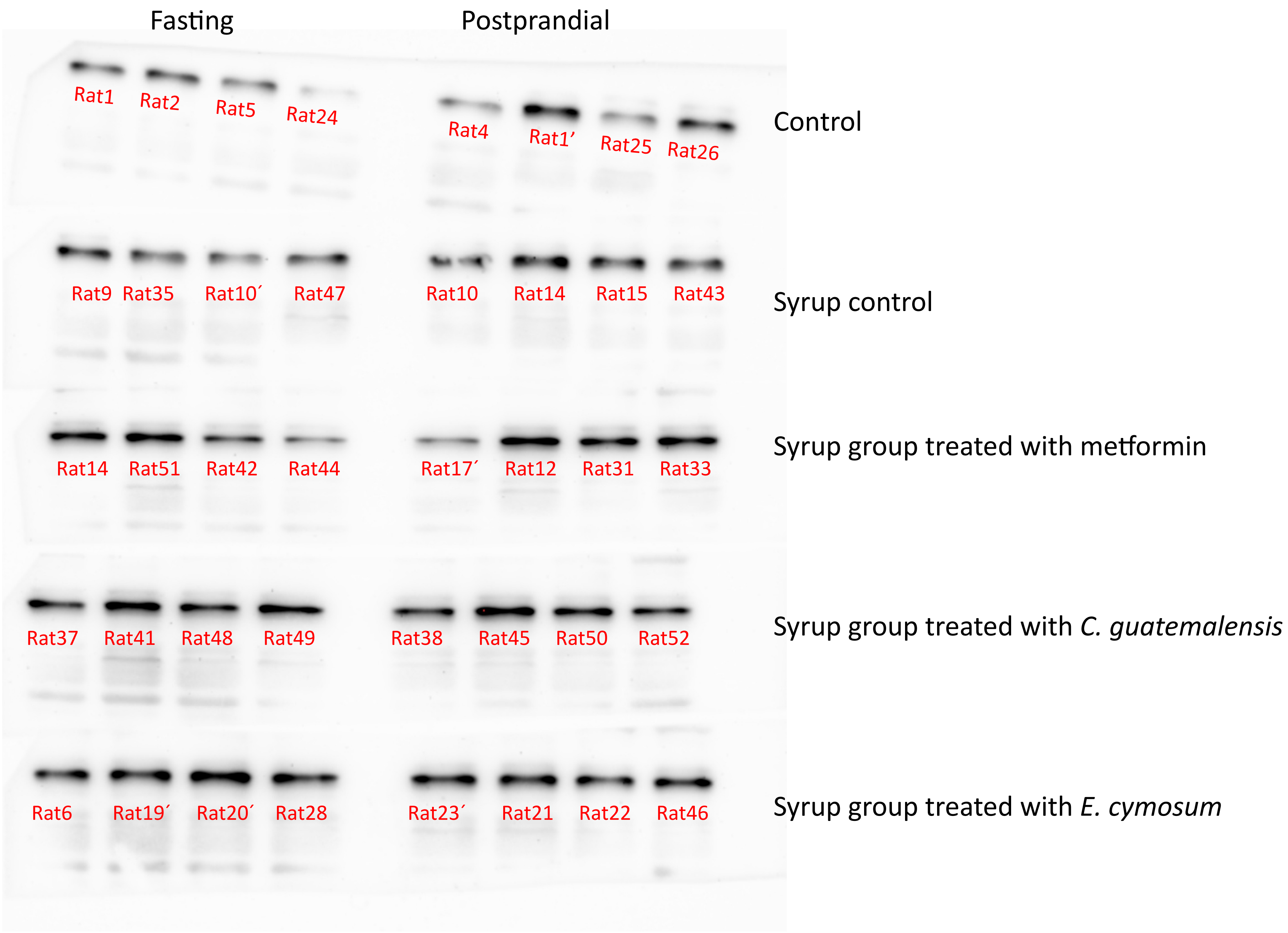

Figure S11. Muscle Blot Akt commasie.

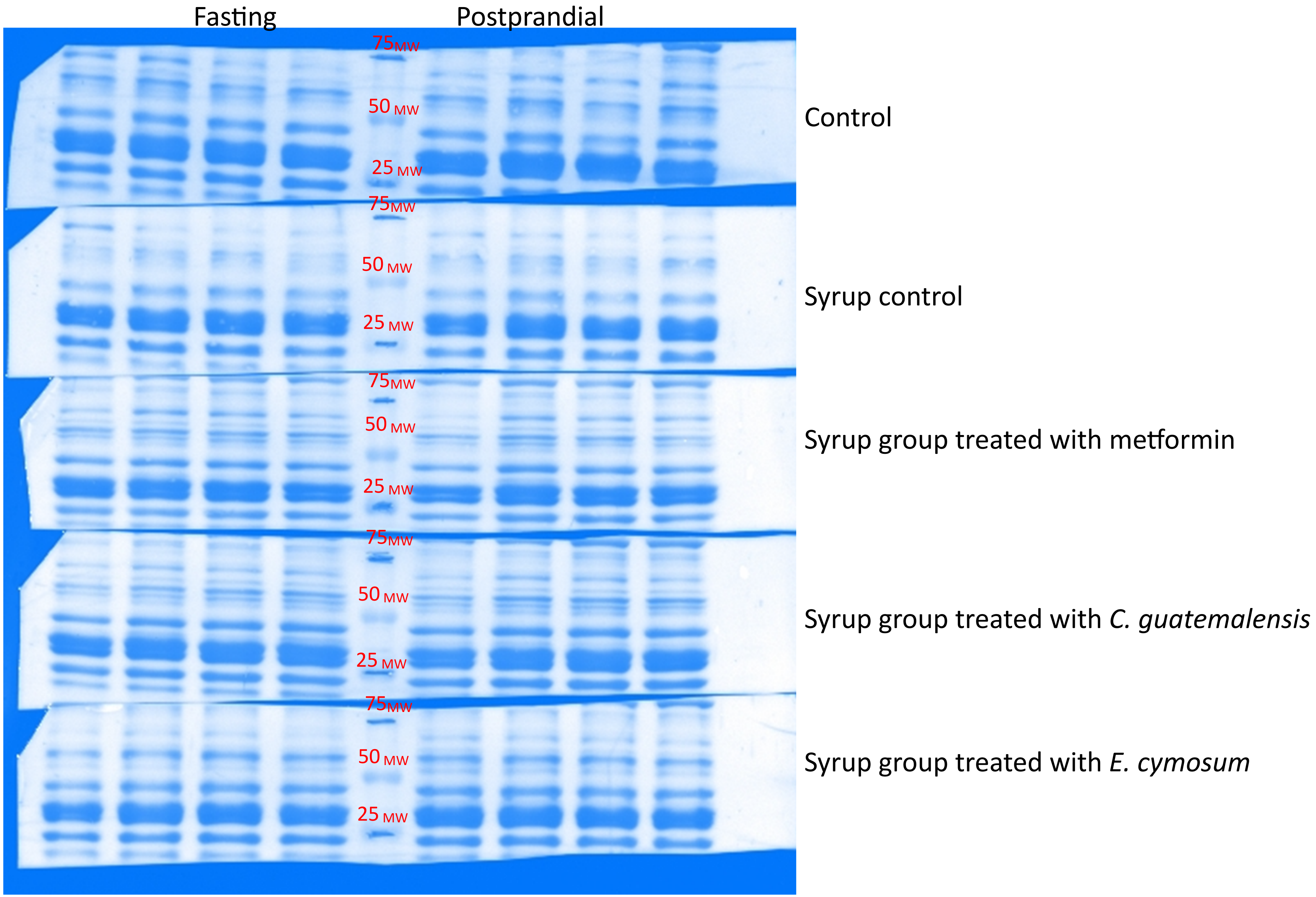

Figure S12. Representative samples Akt muscle MW 62kDa anti-Akt 1:1000.

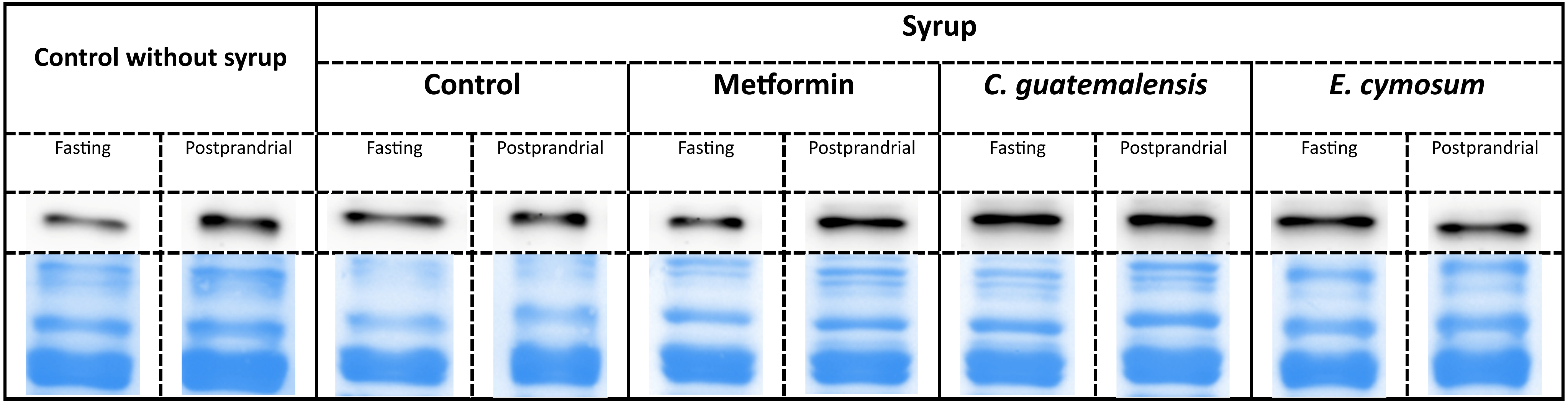

Figure S13. Muscle Blot pAkt1 (Ser 473) chemiluminescence MW 62kDa anti-pAkt 1:500.

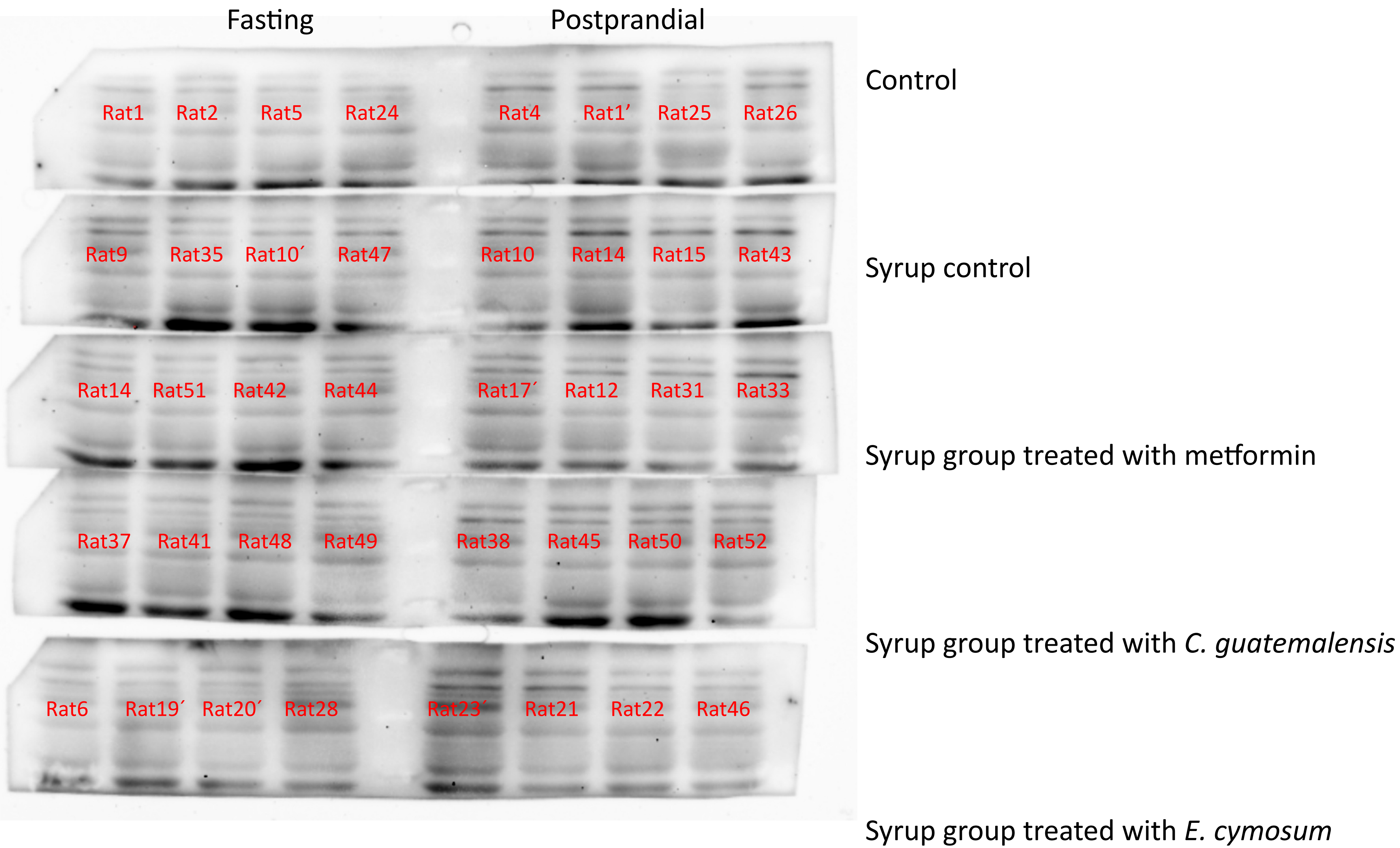

Figure S14. Muscle Blot pAkt1 (Ser 473) commasie.

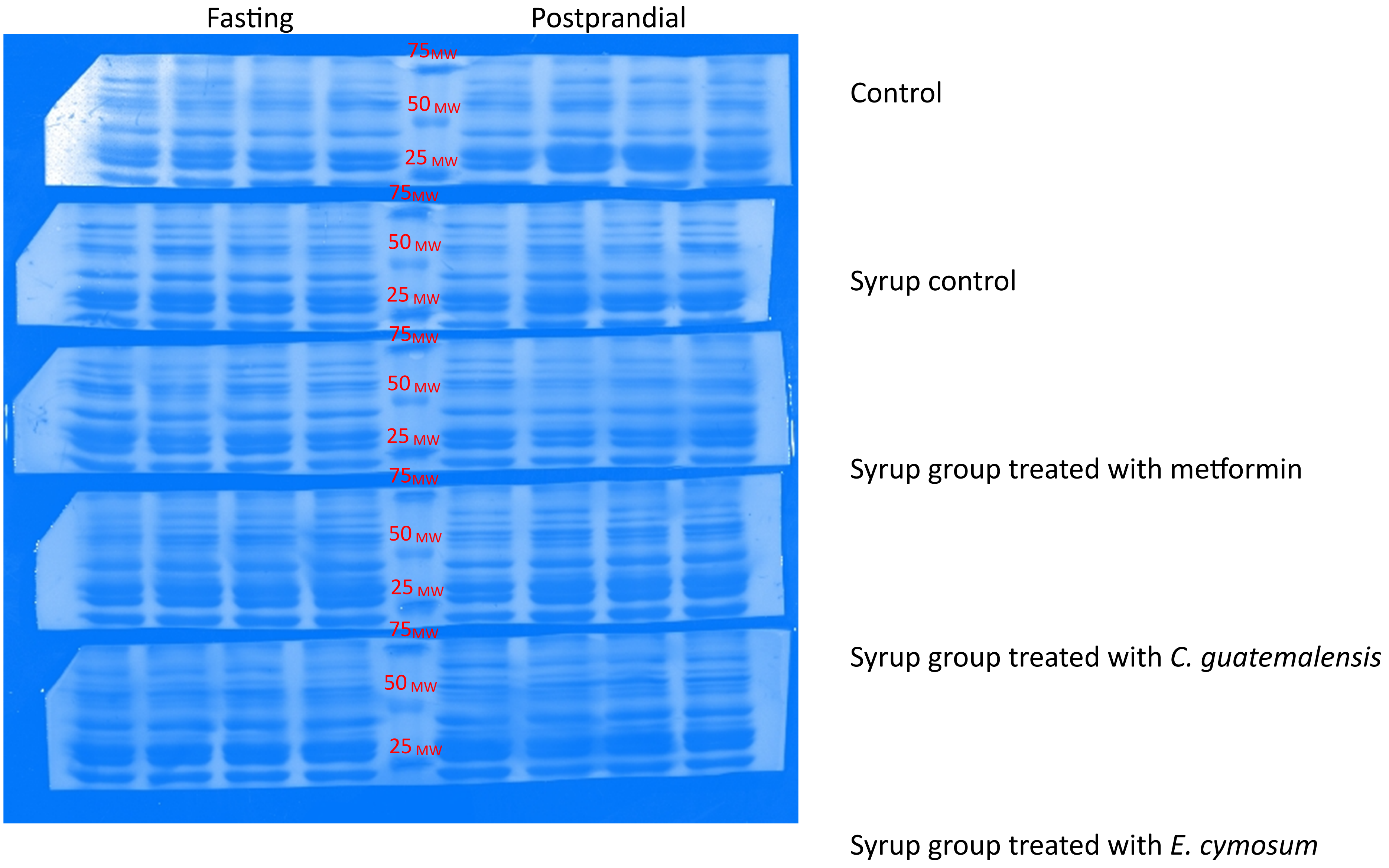

Figure S15. Muscle Representative samples pAkt 1 (Ser 473) MW 62kDa anti-pAkt1 1:500.

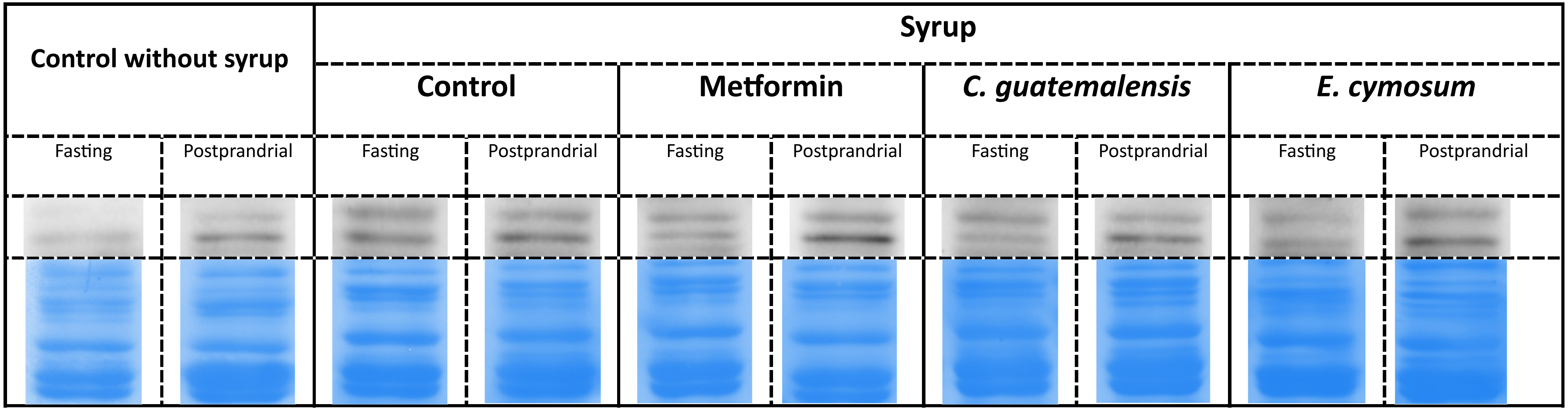

Figure S16. Muscle Blot pAkt2 (Ser 474) chemiluminescence MW 62kDa anti-pAkt2 1:2000.

Fasting Postprandial

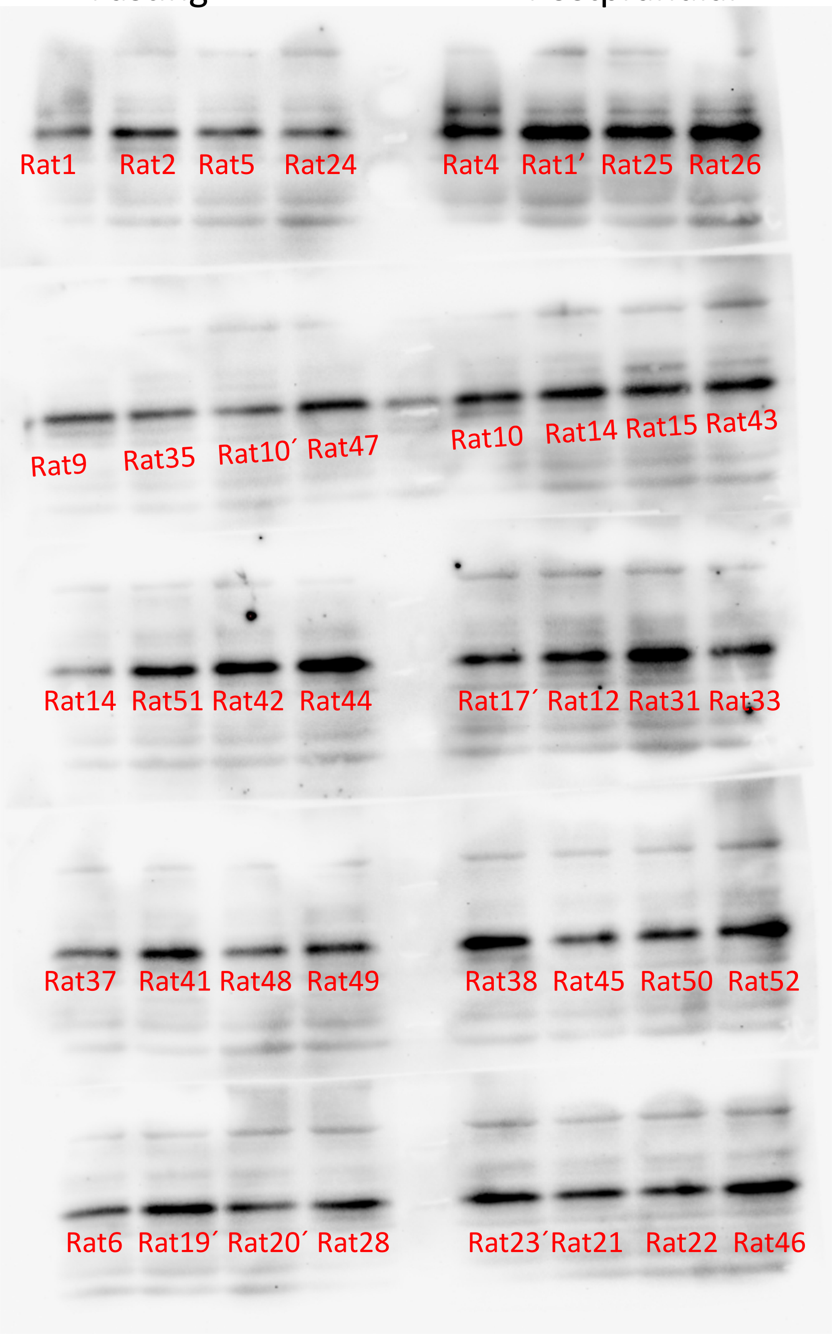

Control

Syrup control

Syrup group treated with metformin

Syrup group treated with *C. guatemalensis*

Syrup group treated with *E. cymosum*

Figure S17. Muscle Blot pAkt2 (Ser 474) commasie.

Fasting Postprandial

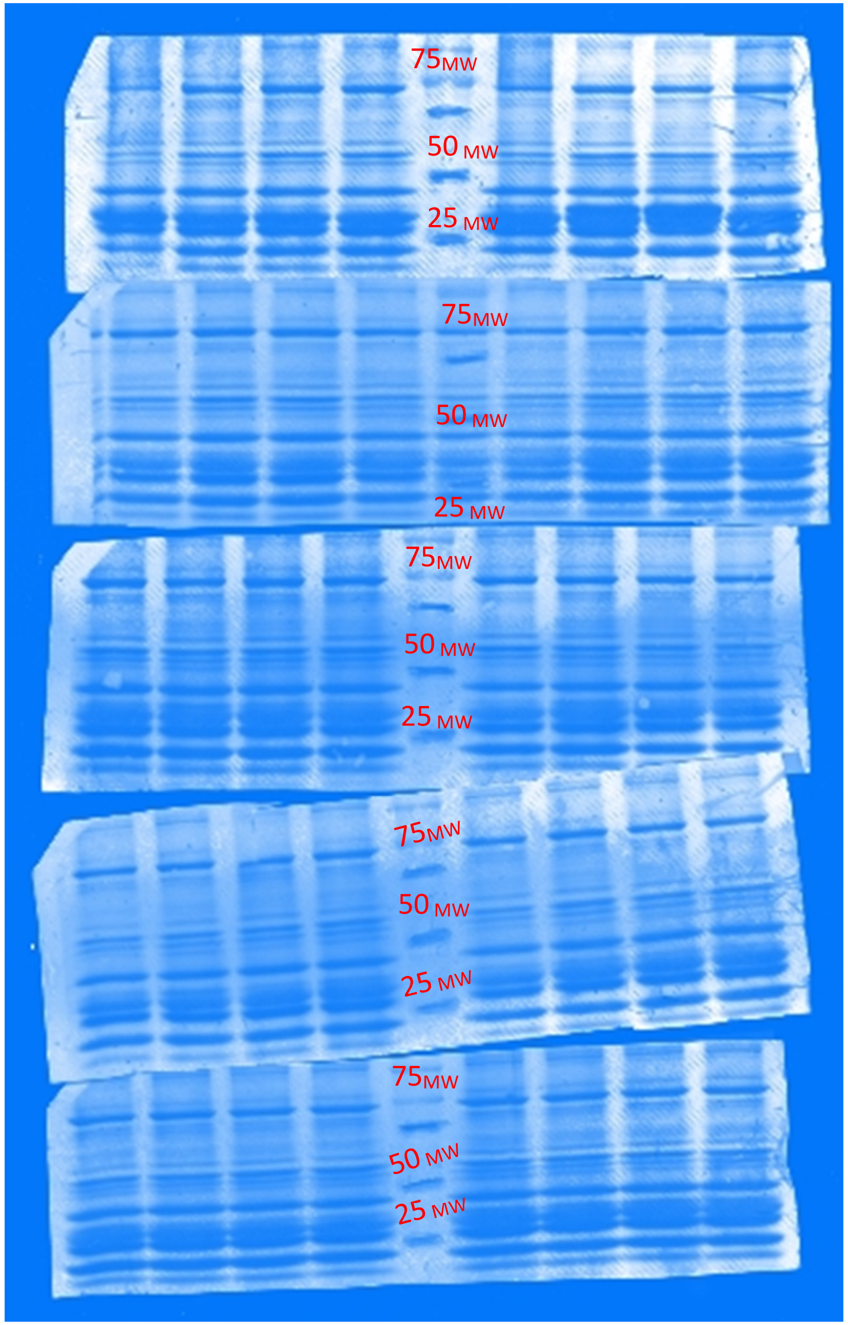

Control

Syrup control

Syrup group treated with metformin

Syrup group treated with *C. guatemalensis*

Syrup group treated with *E. cymosum*

Figure S18. Muscle Representative samples pAkt 2 (Ser 474) MW 62kDa anti-pAkt2 1:2000.

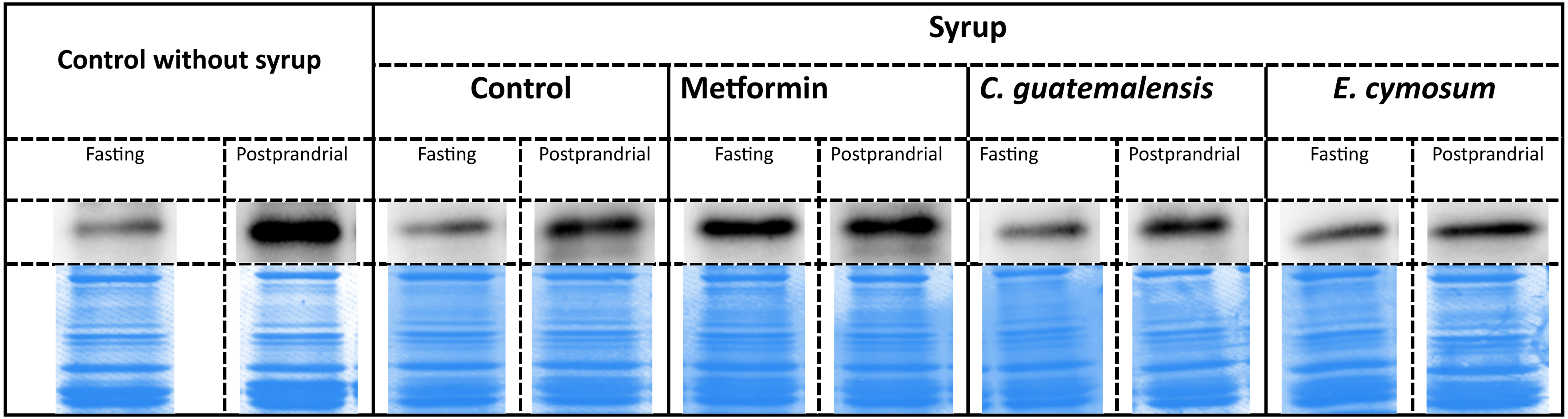

Supplement: Supplementary file 1 [file pharmaceuticals-18-01433-s001.zip › pharmaceuticals-3834012-supplementary.pdf]
